# Laboratory diagnosis of loiasis to support individual patient management: A systematic review

**DOI:** 10.1371/journal.pntd.0014460

**Published:** 2026-07-13

**Authors:** Luzia Veletzky, Marc P. Hübner, Cristina Mazzi, Veronica A. Fittipaldo, Marielle Bouyou-Akotet, Federico Gobbi, Michael Ramharter, Francesca Tamarozzi

**Affiliations:** 1 Department of Medicine I, Division of Infectious Diseases and Tropical Medicine, Medical University of Vienna, Vienna, Austria; 2 University of Bonn, University Hospital Bonn, Institute for Medical Microbiology, Immunology and Parasitology, Bonn, Germany; 3 German Center for Infection Research (DZIF), Partner Site Bonn-Cologne, Bonn, Germany; 4 Clinical Research Unit, IRCCS Sacro Cuore Don Calabria Hospital, Negrar di Valpolicella, Verona, Italy; 5 Department of Infectious-Tropical Diseases and Microbiology, IRCCS Sacro Cuore Don Calabria Hospital, Negrar di Valpolicella, Verona, Italy; 6 Department of Parasitology-Mycology-Tropical Medicine, Université des Sciences de la Santé, Libreville, Gabon; 7 Department of Clinical and Experimental Sciences, University of Brescia, Brescia, Italy; 8 Center for Tropical Medicine, Bernhard Nocht Institute for Tropical Medicine & I. Dep. Medicine University Medical Center Hamburg-Eppendorf, Hamburg, Germany; 9 German Center for Infection Research, Partner Site Hamburg-Lübeck-Borstel-Riems, Germany; 10 Centre de Recherches Médicale de Lambaréné, Lambaréné, Gabon; Washington University in St Louis School of Medicine, UNITED STATES OF AMERICA

## Abstract

**Background:**

Loiasis is a filarial disease caused by *Loa loa*, endemic to Central and West Africa. Cases are observed in migrants and occasionally travellers. Its diagnosis may be difficult due to the unspecific clinical picture and the high percentage of people who do not have microscopically detectable circulating microfilariae. We performed a landscape analysis of the laboratory-based diagnostic techniques available for loiasis and of their estimated sensitivity and specificity.

**Methods:**

We performed a systematic review of cross-sectional, cohort, case-control, diagnostic accuracy, and clinical trial studies published in PubMed, EMBASE, and CENTRAL (searched on March 26^th^ 2025), applying diagnostic assays for human loiasis. When possible, a proportional meta-analysis was performed by estimating sensitivity and specificity separately against eligible reference tests (direct assays or presence of “eyeworm” or their composite or latent class analysis) for each technique category (microscopy of thick smears or concentrated blood, PCR-based or LAMP-based molecular assay, and ELISA-based or rapid -RDT- serological assays). A random-effects model with the DerSimonian-Laird approach was applied. Study quality was evaluated using the Newcastle–Ottawa Scale.

**Results:**

Ninety-nine publications were included in the landscape analysis and 27 were also eligible for performance assessment. Microscopy was applied in 91/99 (91.9%) studies, PCR-based techniques in 29/99 (29.3%), LAMP in 4/99 (4.0%), and serological assays in 19/99 (19.2%). Within techniques categories, characteristics were highly heterogeneous. Sensitivities ranged from 75.0-98.3% for thick blood smears, 48.2-99.9% for blood concentration techniques, 80.6-98.4% for PCR-based techniques, 88.5-98.1% for LAMP-based techniques, 81.9-90.5% for ELISA seroassays, and 43.6-88.0% for RDTs. Cross-reactivity of *L. loa*-specific seroassays with *M. perstans* and other parasitoses was limited (<10%).

**Conclusions:**

Assays for the diagnosis of loiasis are not standardized. ELISA-based serology and possibly PCR-based methods may be appropriate for screening. Microscopy must be performed even in case of negativity on screening when epidemiological or clinical factors suggestive of loiasis are present to plan safe treatment.

## Author summary

Loiasis is an infectious disease caused by the filarial nematode *Loa loa*, endemic to Central and West Africa. In endemic countries, it is estimated that more than 20 million people are chronically infected, with relevant morbidity and mortality. Outside endemic areas, cases are observed in migrants and occasionally in travellers returning from endemic regions. The correct diagnosis of loiasis is of paramount importance to implement the correct treatment in a safe manner. However, infection may be overlooked due to the unspecific clinical picture and, especially outside endemic areas, the often limited knowledge of the infection and low index of suspicion. Furthermore, laboratory diagnostic techniques are not standardized and the diagnosis of infection may be difficult to ascertain in the considerable proportion of individuals that do not have parasite larvae (microfilariae) detectable in blood by microscopy. In preparation for the development of guidelines for the diagnosis and clinical management of patients with loiasis, we performed a systematic review of laboratory-based diagnostics for loiasis to provide a landscape of what are the laboratory-based techniques available, and of their estimated sensitivity and specificity.

## Introduction

Loiasis is a chronic infectious disease caused by the vector-borne filarial nematode *Loa loa*, endemic to Central and West Africa [1]. The parasite is transmitted through the bite of *Chrysops* spp flies. Adult worms reside and migrate in intermuscular fascial layers and subcutaneous tissues, and first-stage larvae (microfilariae - mf), released by adult female worms, circulate in the blood during daytime, where they are taken up by the vector during a blood meal. In endemic countries, it is estimated that more than 20 million people are chronically infected in high or intermediate risk areas (i.e., having prevalence of loiasis higher than 20%) [1], causing significant morbidity and mortality [2–4]. Outside endemic areas, cases are not infrequently observed in migrants and occasionally in travellers who have been infected in endemic regions [5–13]; however, there are no credible statistics about the number of imported cases.

Typical clinical manifestations are the migration of the adult worm under the conjunctiva (“eyeworm”) and episodes of transient localized oedema (“Calabar swelling”), which are reported by a very variable percentage of infected subjects (5–66% and 17–91%, respectively) [2,6–8,10–12,14–19]. However, infection can range from being asymptomatic to being accompanied by a variety of unspecific signs and symptoms [1,20–22]. In addition, severe, even fatal, neurological adverse events may occur after administration of antifilarial drugs or even spontaneously in individuals with high *L. loa* blood mf loads [22–24].

The correct diagnosis of loiasis at an individual level, as opposed to population-based prevalence estimate for public health purposes, is of paramount importance to implement the correct treatment aiming at parasitological cure and subsequent follow-up. However, diagnosis of infection may be difficult to ascertain due to the unspecific clinical picture and, especially outside endemic areas, the often limited knowledge of the infection and low index of suspicion.

Diagnostic procedures for loiasis are not standardized. In clinical routine, diagnosis classically relies on the identification of *L. loa* mf in peripheral blood samples by microscopy, either in thick blood smears or after concentration techniques using various methods such as filtration or leukoconcentration. Additionally, molecular assays such as PCR or Loop-mediated isothermal amplification (LAMP) have been developed. However, in a variable proportion of infected subjects, estimated generally between 40–65% [1,25], no circulating mf can be detected, a state that is categorized as amicrofilaraemic and traditionally referred to as “occult loiasis”, making diagnostic confirmation difficult.

Other possible and mostly indirect diagnostic tools comprise a variety of serological techniques which have been described in the literature and assessed for accuracy; however, these assays are mostly non-standardized in-house tests and no *L. loa*-specific commercial molecular or serological assays are currently available. One lateral flow rapid diagnostic test (RDT) has been developed by Drugs & Diagnostics for Tropical Diseases (DDTD, San Diego, CA, USA) and assessed in studies, but the test has not been marketed [26].

In the process of evidence assessment for the preparation of the guidelines for the diagnosis and clinical management of individual patients with loiasis, we performed a systematic review of laboratory-based diagnostics for loiasis to provide a landscape of what techniques are available, and of their estimated sensitivity and specificity.

## Materials and methods

### Search strategy and selection criteria

The study protocol was registered with PROSPERO (CRD420251047013). The work is presented according to the recommendations of the Preferred Reporting Items for Systematic Reviews and Meta-Analyses of Diagnostic Test Accuracy studies (PRISMA-DTA) [27] (S1 File).

Eligible studies were cross-sectional, cohort, case-control, diagnostic accuracy, and clinical trial studies published in peer-reviewed journals. Manuscripts reporting case reports and reviews were not eligible, and original publications from which the original data could not be extracted were also excluded. For the landscape analysis of applied diagnostic techniques, all studies applying diagnostic assays with the aim of detecting *L. loa* infection in humans were included. For performance assessment and, when possible, meta-analysis, the subset of these studies comparing two or more tests were included. Publications addressing diagnostic target discovery only were excluded.

The target population was composed by individuals of all ages and both sexes, where infection with *L. loa* was assessed by microscopy or molecular analysis or “eyeworm” either witnessed by health personnel or identified as described by the WHO guidelines for rapid assessment of *Loa loa* (RAPLOA [28]). Eligible reference tests were direct microscopy-based or molecular assay or “eyeworm” identification as described above, or composites of these methods, or latent class analysis (LCA) encompassing these methods. Index tests were defined as any laboratory-based assay for the diagnosis of loiasis. Studies or groups of individuals within a study for which the diagnosis was based on clinical signs/symptoms or methods other than those detailed above were excluded, with the exception of uninfected controls who had never travelled/resided in *L. loa* endemic areas, who were considered *bona fide* uninfected. No restriction was applied regarding the study setting (endemic or non-endemic area; population or clinical setting).

The literature search was conducted on March 26^th^ 2025 in PubMed (MEDLINE), EMBASE, and Cochrane Central Register of Controlled Trials (CENTRAL). The search strategy is reported in S2 File. No publication date or language limitations were applied. Neither Google Scholar or other sources of grey literature, nor the bioRxiv.org portal, were searched. Duplicates were removed before screening for relevance by title and abstract. Although reviews were excluded, their bibliography list, as well as the bibliography lists of the included papers, were examined for additional relevant papers. Each publication was evaluated in parallel by two authors among the three who conducted the selection and evaluation (LV, MPH, FT). Retrieved records were screened by title and abstract using the Rayyan online platform [29], and the full text of potentially eligible papers retrieved and data extracted independently by two authors using a pre-set Microsoft Excel spreadsheet. At all steps, disagreements between authors were resolved by discussion with the support of the third author.

Data extracted were: 1) paper references (first and last author, year of publication, DOI/PMID); 2) country/ies where the study was carried out; 3) study design; 4) setting (clinical/population); 5) definition of cases and controls; 6) diagnostic test(s) applied; 7) if eligible for sensitivity and specificity data extraction, what reference and what index tests were applied; 8) for each index test, number of true positive, true negative, false positive, and false negative samples compared to all eligible reference tests. The latter figures were extracted as reported in the paper (i.e., for seroassays with a cut-off, no calculations were done by changing thresholds for positivity) or calculated from sensitivity, specificity, or predictive values, when possible, if raw numbers were not available from the paper or supplementary materials. If calculated, figures were rounded at the lower or higher integer in case decimals obtained were <0.5 or ≥0.5, respectively.

### Data analysis

For the analysis, assays were grouped by category: microscopy of thick smears (any blood volume), microscopy of concentrated blood (any blood volume and any concentration technique), PCR-based molecular assay (any *L. loa*-specific target and PCR technique), LAMP-based molecular assay (any *L. loa*-specific target), ELISA-based antibody (Ab)-detecting serological assay (based on any antigen and capturing any Ab type), and RDT-based Ab-detecting serological assay (based on any antigen and capturing any Ab type). Techniques other than those belonging to these categories were only described.

Given the diagnostic accuracy design of most studies (16/27; 59.3%), the high percentage (10/27 (37%) of studies where infected and uninfected individuals were not derived from comparable groups of individuals, and the considerable heterogeneity of reference standard used of most included studies, we conducted a proportional meta-analysis by estimating sensitivity and specificity independently.

Sensitivity and specificity were assessed separately against all eligible reference tests for each technique category. When possible, sensitivity was stratified by occult/non-occult loiasis. Since “occult loiasis” is not univocally defined, for the purpose of this analysis “occult loiasis” was defined as an individual with “eyeworm” in a patient with no microfilariae on microscopy (amicrofilaraemic) in the clinical context (independent of molecular positivity), and as amicrofilaraemic infection identified through the RAPLOA questionnaire in the population context. Specificity was assessed against the reference standard category “*L. loa*-negative by reference test“; however, when possible, specificity was also assessed stratified by control type: non-endemic controls, *L. loa*-negative but positive for *Mansonella perstans* or the sympatric sibling species *Mansonella* “DEUX” [30] (later referred to as *M. perstans* cumulatively), both *L. loa*-negative and *M. perstans*-negative, and other helminthiases.

A random-effects model with the DerSimonian-Laird approach and with the variance-stabilizing Freeman-Tukey double arcsine transformation was used to account for the expected heterogeneity between studies. Forest plots were used to visualize the effect sizes and 95% confidence intervals (CI) of individual studies. The effects were estimated using the inverse variance method and the confidence intervals were calculated using the Wilson score formula with continuity correction. Heterogeneity was assessed using Cochran’s Q test, with I2 ≥ 50% indicating statistical heterogeneity. Analyses were performed using the Metafor [31] and Meta [32] package in R version 4.5.0.

Quality evaluation was carried out only for studies included in the performance assessment analysis. An adapted version of the Newcastle – Ottawa quality assessment Scale (NOS) [33] was used (S3 File). Three main domains were used: selection of studies, comparability, and outcome. The selection domain included study design, reference diagnostic test, definition of controls, and evaluation of cross-reactivity; the comparability domain included comparability of cases and controls; and the outcome domain included blinding regarding results of the reference assay by personnel implementing the index test.

## Results

### Data retrieval

**Fig 1:**
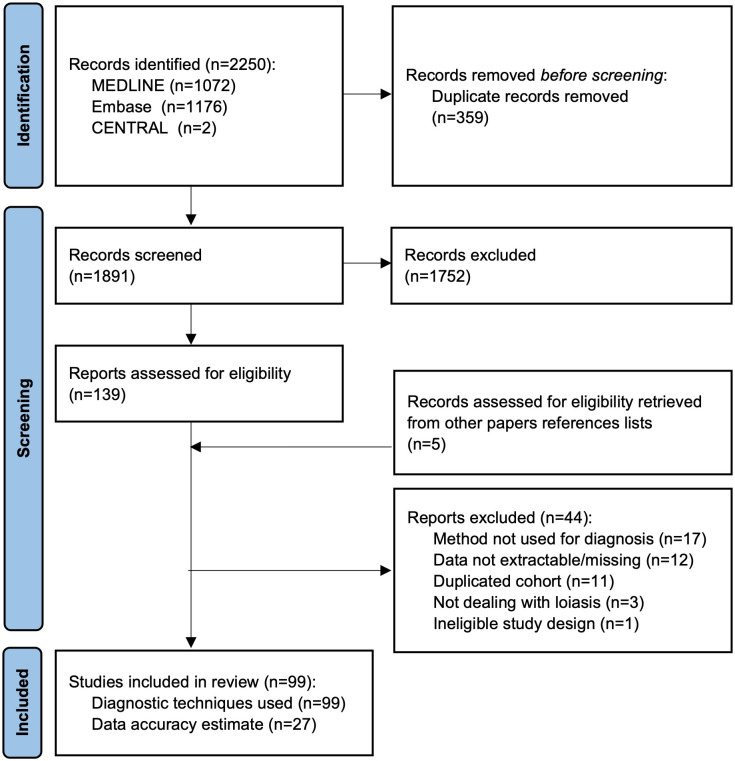
Flow chart of the study identification, screening, and inclusion procedure.

The literature search identified 1891 publications. After screening by title and abstract, 139 potentially eligible publications were shortlisted for full text evaluation. Of these, 95 publications were included for the landscape analysis of laboratory-based techniques available to diagnose human infection with *L. loa*. Further five potentially eligible publications were retrieved from the list of references of included publications, of which four were included, for a total of 99 publications included for the landscape analysis. Of these 99 publications, 27 were also eligible for the extraction of performance data and quality assessment. The flow chart of the studies identification, screening and inclusion is presented in Fig 1. The database containing the complete list of included publications, data extraction and quality assessment is available as S4 File.

### Landscape of assays used for the diagnosis of loiasis

Of the 99 publications included for the landscape analysis of laboratory-based techniques applied or evaluated to diagnose human infection with *L. loa,* 80 (80.8%) described studies conducted in endemic areas. The majority of included studies from endemic areas had a cross-sectional (50/80, 62.5%) design and was population-based (64/80, 80%), while studies in non-endemic areas were mainly diagnostic accuracy (8/19, 42.1%) and cohort studies (5/19, 26.3%) and were conducted in a clinical/laboratory setting (14/19, 73.7%). The synthesis of study settings, designs and diagnostic methods used is presented in Table 1.

**Table 1:** Synthesis of study settings, designs and diagnostic methods used in the included publications for the laboratory-based diagnosis of loiasis.

	Studies in endemic areas (N = 80)	Studies in non-endemic areas (N = 19)	Total (N = 99)
**Study design [N (%)]**			
- Cross-sectional	50 (62.5%)	2 (10.5%)	52 (52.5%)
- Diagnostic accuracy	10 (12.5%)	8 (42.1%)	18 (18.2%)
- Cohort	8 (10.0%)	5 (26.3%)	13 (13.1%)
- Case-control	3 (3.8%)	1 (5.3%)	4 (4.0%)
- Other*	9 (11.25%)	3 (15.8%)	12 (12.1%)
**Study setting [N (%)]**			
- Population-based	64 (80.0%)	5 (26.3%)	69 (69.7%)
- Clinical/Laboratory	16 (20.0%)	14 (73.7%)	30 (30.3%)
**Diagnostic method [N (%)]** ^**§**^			
**-** Thick blood smear	53 (66.3%)	8 (42.1%)	61 (61.6%)
- Mf concentration method^	26 (32.5%)	9 (47.5%)	35 (35.4%)
- NAAT	21 (26.3%)	8 (42.1%)	29 (29.3%)
- Serological assay	13 (16.3%)	7 (36.8%)	20 (20.2%)
- LoaScope°	6 (7.5%)	–	6 (6.1%)
- Other”	–	2 (10.5%)	2 (2.0%)

Mf = microfilariae. NAAT = Nucleic Acid Amplification Tests (include PCR-based methods and LAMP). *Other study designs include clinical trials, immunology studies, and technical assessment studies. ^§^Single studies might have included more than one diagnostic method, therefore sums may exceed 100%. °Smartphone-based microscope that automatically detects and quantifies microfilariae in blood [34]. ^All studies in endemic areas used leukoconcentration; four of the studies carried out outside endemic areas concentrated the mf using filtration, while five used leukoconcentration. “Scepter 2.0 repurposed hand-held automated cell counter (Millipore).

**Fig 2:**
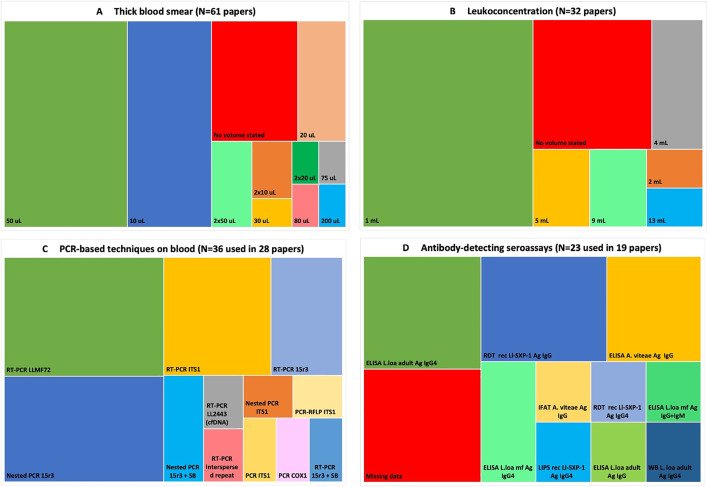
Treemap chart of the landscape of the characteristics of assays described for the diagnosis of loiasis. Each box represents an assay category; the size of each box is proportional to the frequency of use in the included papers**. A)** Blood volume used to perform thick blood smears. **B)** Blood volume used to perform leukoconcentration. **C)** PCR-based techniques and targets performed on blood or blood-derived specimens. The paper describing PCR from feces was not included in the graph. **D)** Antibody-detecting serological assay formats and design. RFLP = restriction fragment length polymorphism. SB = Southern Blot. RDT = rapid diagnostic test. IFAT = immunofluorescence antibody test. LIPS= luciferase immunoprecipitation system. WB = Western Blot. Ag = antigen. Mf = microfilariae.

At least one microscopy technique for the identification of mf was applied in the majority of studies (91/99, 91.9%). Thick blood smears were applied in 61 (61/99; 61.6%) publications, while an mf concentration technique was applied in 35 (35/99; 35.4%) publications. Microscopy on thick blood smears was performed in 66.3% (53/80) of studies carried out in endemic areas, and after concentration techniques in 47.5% (9/19) of studies conducted in non-endemic areas. There was a high heterogeneity in the volume of blood used for both of these technique categories (Fig 2A). Thick blood smears were prepared from 10 to 200 µL blood, the most frequently used being a single smear of 50 µL (22/61 publications, 36.1%). The staining method was reported in 44/61 (72.1%) publications, and was Giemsa in the majority of studies (39/61, 63.9%), followed by no staining (4/61, 6.6%) and other staining (Leishman or Field staining, one publication in each case). The most frequently used mf concentration technique was leukoconcentration (32/35, 91.4%), while blood filtration was used only in 4/35 studies (11.4%). For leukoconcentration, the starting volume of blood was highly heterogeneous (Fig 2B), ranging from 1 to 13 mL, the most frequently used being 1 mL (16/35 publications, 45.7%). Filtration was always carried out on 1 mL blood. Staining after mf concentration was only reported in 8/35 (22.9%) publications, being Giemsa in six cases, Carazzi haematoxylin in one case, and methylene blue in one case. Other mf-detecting techniques described in the included publications were the LoaScope [34], a smartphone-based microscope that automatically detects and quantifies mf in blood, used in 6/99 (6.1%) studies, and the Scepter 2.0 repurposed hand-held automated cell counter from Millipore (Billerica, MA, USA) [35] used in two studies (2/99; 2.0%).

Molecular detection methods included PCR-based assays and LAMP. All molecular assays were in-house. One or more PCR-based techniques were performed in 29 (29/99; 29.3%) studies (Fig 2C): real-time PCR was the most common technique used (20/29; 69.0%). The most frequent targets were the repeat-3 sequence of the 15-kDa *L. loa* polyprotein gene (15r3) [36], used in 11/29 (37.9%) publications, and the LLMF2 gene [37], used in 9/29 (31.0%) publications. DNA for the applied reactions was extracted from blood or a blood-derived matrix in 28/29 (96.5%) publications. DNA was extracted either from blood (18/29, 62.1%) or dried blood spots (8/29; 27.6%) in the majority of cases. In one case, plasma was used to detect cell-free DNA [38], in another the DNA was obtained from filters of RDTs for malaria [39], and in a third publication, DNA was extracted from faeces [40]. LAMP on one or more targets was performed only in four (4/99; 4.0%) studies. LAMP targeted the LLMF72 gene in two studies [41,42], the LLMF342 gene [42], the RF4 family repeat [43], and an 839 bp repetitive sequence in one study each, respectively [44].

Finally, one or more serological assay was used in 20/99 (20.2%) studies, encompassing ELISA (used in 14 studies), immunochromatographic RDT test (5 studies), indirect immunofluorescence (2 studies) and western blot and luciferase immunoprecipitation system formats (1 study each) (Fig 2D). In the 20 publications describing seroassays, 24 assays were applied: of these, in all but one case (23/24 assays), the assays used were designed to detect human antibodies; in only one study [45] an antigen-capturing ELISA assay was applied. In the majority of serology assays detecting antibodies (13/23 of antibody-detected assays described; 56.5%), the assay was produced in-house and, of these, ELISA based on adult *L. loa* antigen detecting IgG4 was the most frequent design (4/13; 30.8%). The RDT based on the *L. loa* recombinant Ll-SXP-1 antigen [26,46] produced by Drugs & Diagnostics for Tropical Diseases (San Diego, CA, USA) as a Research-Use-Only test, was used in 5/20 (25.0%) studies, most frequently in the version detecting IgG. The only commercial assay applied was the pan-filarial *Acanthocheilonema viteae* IgG ELISA (Bordier Affinity Products, Crissier, Switzerland), used in three studies carried out in the clinical setting outside endemic areas.

### Performance assessment: sensitivity

**Fig 3:**
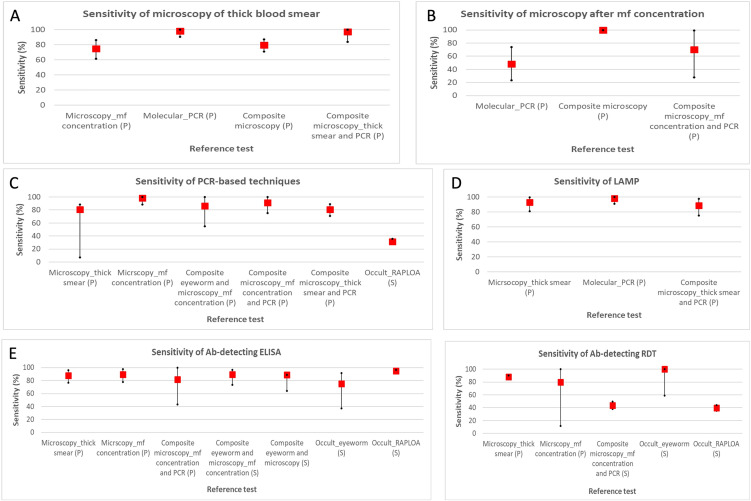
Summary of sensitivity (Y-axis) of diagnostic techniques categories according to reference standard used (X-axis). The graph summarizes the results of forest plots available as S5 File, by plotting summary measures of the forest plots. Red boxes represent pooled sensitivity if obtained from the results of multiple comparisons (P) or sensitivity when obtained from the results of a single comparison (S). Error bars depict 95%CI.

Of the 21 studies included in the meta-analysis for sensitivity (S5 File), only four (19.0%) were carried out outside endemic areas using samples not obtained in endemic areas. However, a separate analysis of the results of these studies compared to those carried out in endemic areas or on samples obtained from individuals in endemic areas was not performed due to the heterogeneity of the tests compared in each study and the impossibility to extract data separately from individuals who came from endemic areas (i.e., continuously exposed to *L. loa*) and travellers (i.e., only temporarily exposed). A summary of ranges of the results of the sensitivity of diagnostic techniques categories for the diagnosis of loiasis is presented in Table 2 and Fig 3.

**Table 2:** Summary of sensitivity ranges of diagnostic techniques categories. The table shows the minimum and maximum estimated sensitivity for each assay category, and the reference test against which the sensitivity was measured. “[Pooled/single]” indicates whether the reported sensitivity was pooled from the results of multiple comparisons or obtained from the results of a single comparison.

Assay	Minimum sensitivity		Maximum sensitivity	
	Reference test	Se (95%CI) [pooled/single]	Reference test	Se (95%CI) [pooled/single]
**Thick blood smear**	Microscopy, mf concentration technique	75.0% (61.4-86.6) [pooled]	Molecular, PCR-based	98.3% (90.7-100) [pooled]
**Mf concentration**	Molecular, PCR-based	48.2% (23.1-73.7) [pooled]	Microscopy-based composite standard	99.9% (96.3-100) [pooled]
**PCR-based techniques**				
**Lab-based ref assays**	Composite: Microscopy thick smear + PCR	80.6% (70.7-89.1) [pooled]	Microscopy, mf concentration technique	98.4% (88.7-100) [pooled]
Occult loiasis*	RAPLOA	31.5% (27.6-35.8) [single]	Eyeworm and/or PCR-positivity but mf- on leukoconcentration	78.1% (19.6-100) [pooled]
**LAMP**	Composite: Microscopy thick smear + PCR	88.5% (75.3-97.6) [pooled]	Molecular, PCR-based	98.1% (90.9-100) [pooled]
**Serology Ab-ELISA**				
**Lab-based ref assays**	Composite: Microscopy mf concentration technique + PCR	81.9% (43.5-100) [pooled]	Microscopy, mf concentration technique	90.3% (79.4-97.6) [pooled]
Occult loiasis*	Eyeworm but mf- on leukoconcentration	75.0% (37.0-91.4) [single]	RAPLOA	94.8% (92.4-95.5) [single]
**Serology Ab-RDT**				
**Lab-based ref assays**	Composite: Microscopy mf concentration technique + PCR	43.6% (38.0-49.4) [single]	Microscopy, thick blood smear	88.0% (85.4-90.4) [pooled]
Occult loiasis*	RAPLOA	39.4% (35.2-43.8) [single]	Eyeworm but mf- on leukoconcentration	100% (58.8-95.0) [single]

Mf = microfilariae. RDT = immunochromatographic rapid test (all based on recombinant Ll-SXP-1 antigen). Ab-ELISA = Antibody-detecting ELISA. Ab-RDT = Antibody-detecting RDT. *Since “occult loiasis” is not univocally defined, for the purpose of this analysis “occult loiasis” was defined as an individual with history of observed “eyeworm” migration in a patient with no microfilariae on microscopy (amicrofilaraemic) in the clinical context (independent of molecular positivity), and as amicrofilaraemic infection identified through the RAPLOA questionnaire in the population context.

#### Microscopy-based techniques.

Pooled sensitivity of thick blood smears ranged from 75.0% (95% CI 61.4-86.6%) when the reference standard was an mf concentration technique, to 98.3% (95% CI 90.7-100%) when the reference standard was a PCR-based method (Table 2, Fig 3A and S5 File). One publication [47] evaluated the performance of Field’s stain, a rapid (1 min) version of the Romanowsky staining method originally developed to detect *Plasmodium* parasites, as a potential rapid staining technique for blood mf compared to the more time-consuming (30 min) Giemsa method, finding it equally suitable. Also, one publication [48] compared the use of capillary and venous blood for the detection of mf by thick blood smears, reporting higher mf counts in capillary blood.

The pooled sensitivities of mf concentration techniques ranged from 48.2% (95%CI 23.1-73.7%) when the reference test was a PCR-based technique, to 99.9% (95%CI 96.3-100%) when the reference was a composite encompassing multiple microscopy techniques (Table 2, Fig 3B and S5 File). In studies performed outside endemic areas using samples obtained outside endemic areas (i.e., migrants and travellers), sensitivities tended towards the upper range of the pooled results when mf concentration techniques were compared to PCR as the reference test (S5 File). These figures are lower than those obtained for thick blood smears, which was unexpected since blood concentration techniques use about x1000 more blood volume. However, it must be highlighted that these sensitivity estimates derived from the analysis of a different set of publications. Actually, when considering the only two studies [49,50] comparing directly thick blood smears and mf concentration techniques using the same reference standard, the pooled sensitivity of mf concentration techniques was higher (99.9%, 95%CI 96.3-100%) than that of thick blood smears (79.9%, 95%CI 71.2-87.1%) (S5 File).

#### Molecular techniques.

The pooled sensitivity of PCR-based techniques performed on blood ranged from 80.6% (95%CI 70.7-89.1%) when the reference standard was a composite of microscopy of thick blood smears and PCR, to 98.4% (95%CI 88.7-100%) when the reference standard was microscopy after blood concentration (Table 2, Fig 3C and S5 File). When considering sensitivity of PCR-based techniques in cohorts of occult loiasis (S5 File), this ranged from 31.5% (95%CI 27.6-35.8%) in a study using RAPLOA as the reference diagnostic [51], to 78.1% (95%CI 19.6-100%) in a study using a composite reference including documented eyeworm and/or PCR-positivity but negative microscopy as the reference standard [52]. One paper [38] investigated cell-free DNA from plasma in a diagnostic accuracy study performed on 53 microscopy-confirmed, archived samples, finding that 48/53 (90.6%) samples were positive.

Sensitivities of LAMP assays were overall comparable to those of PCR-based techniques, ranging from 88.5% (95%CI 75.3-97.6%) when the reference was a composite of microscopy on thick blood smear and PCR, to 98.1% (90.9-100%) when the reference was PCR (Table 2, Fig 3D and S5 File). Unfortunately, a separate evaluation of LAMP for microscopically amicrofilaraemic loiasis was not possible.

#### Serology-based techniques.

Overall, when compared to direct diagnostic tests (microscopy, PCR) as a reference, the sensitivity of antibody-detecting ELISA assays ranged from 81.9% (95%CI 43.5-100%) when compared to a reference encompassing mf concentration techniques and PCR, to 90.5% (95% CI 78.7%-98.4%) when the reference test was microscopy on thick blood smear (Table 2, Fig 3E and S5 File). When considering occult loiasis, sensitivity of antibody-detecting ELISA assays ranged from 75.0% (95%CI 37.0-91.4%) when infection was clinically assessed by eyeworm observation, to 94.8% (95%CI 92.4-96.5%) when defined by RAPLOA (Table 2, Fig 3E and S5 File).

Interestingly, the range of sensitivity of the recombinant Ll-SXP-1-based RDT assay was wider than that reported for ELISA assays, from 43.6% (95%CI 38-49.4%) when the reference was a composite of microscopy after mf concentration and PCR, to 88.0% (95% CI 85.4-90.4%) when the reference was microscopy on thick blood smear (Table 2, Fig 3F and S5 File). The same was observed for occult loiasis, where sensitivity of the RDT ranged from 39.4% (95%CI 35.2-43.8%) when this was defined by RAPLOA, to 100% (95%CI 58.8-95%) when defined by eyeworm observation (Table 2, Fig 3G and S5 File). Only one study [53] directly compared the IgG-capturing and IgG4-capturing versions of the RDT, reporting no different sensitivities (S5 File).

Only one study [51] applied in parallel a *L. loa*-specific IgG ELISA based on adult worm antigen and the IgG-detecting recombinant Ll-SXP-1-based RDT finding that, when compared to the same reference assays, the former had sensitivities of 94.3-94.8% while the range of sensitivities of the latter was between 39.4-44.1%.

The sensitivity for loiasis estimated from the only eligible study applying a commercial pan-filarial ELISA based on antigens from *A. viteae* [54] was in line with those obtained with ELISA assays based on *L. loa* antigens for both composite reference standard of microscopy concentration technique and “eyeworm” (89.2%; 95%CI 73.6-96.5%) and occult loiasis (75%; 37.0-91.4%)

Finally, one study [45] evaluated an antigen-capture ELISA detecting the Ll-Bhp-1 antigen using a sample cohort of 116 sera from patients diagnosed by 50 ul thick blood smear. Reported sensitivity was 63.8%.

### Performance assessment: specificity

Of the 14 studies included in the meta-analysis for specificity (S6 File), only three (21.4%) were carried out outside endemic areas using samples not obtained in endemic areas. However, a separate analysis of the results of these studies compared to those carried out in endemic areas or on samples obtained from individuals in endemic areas was notperformed due to the heterogeneity on the tests compared in each study and the impossibility to extract data separately from individuals who came from endemic areas (i.e., continuously exposed to *L. loa*) and travellers (i.e., only temporarily exposed).

#### Serology-based techniques.

**Fig 4:**
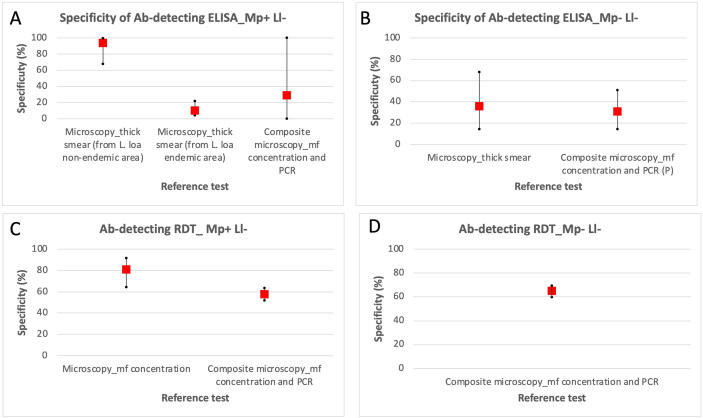
Summary of specificity (Y-axis) of Ab-based serological assays according to direct (microscopy and/or molecular) reference standard used (X-axis) for *L. loa* and *M. perstans* diagnosis. The graph summarizes the results of forest plots available as S6 File, by plotting summary measures of the forest plots. Red boxes represent specificity, error bars depict 95%CI. P = pooled specificity (all other figures are derived from a single studies). Ab = antibody. Mp = *Mansonella perstans*. Ll = *Loa loa*.

No false-positive results were reported in studies assessing specificity of *L. loa*-specific or pan-filarial antibody-detecting ELISA or RDT assays on sera from healthy individuals from non-endemic areas (non-endemic controls) (S6 File). However, different levels of cross-reactivity were reported for *L. loa*-specific antibody-detecting serology assays with sera from patients with other parasitic infections (S6 File). One study evaluating the IgG-detecting Ll-SXP-1-based RDT [54] found a specificity of 97.9% (95%CI 87.3-99.9) on a cohort of sera from patients with different non-filarial parasitoses and no known exposure to loiasis. In this study, for which breakdown of cross-reactivity with each parasite type was available, the only false-positive result (1/47 samples) was obtained in a serum from a patient with *Schistosoma* infection and without reported travel to *L. loa* endemic areas (but possible contact with other filariae was not specified). Another study evaluating an ELISA detecting IgG against an adult *L. loa* crude extract [51] found a specificity 43.9% (95%CI 28.8-60.1) on a cohort of sera from patients with different filarial and non-filarial parasitoses, but unfortunately break-down of data by parasite species was not available. In this study, five of the 41 sera of the “other parasitoses” cohort were from individuals with mansonellosis, a common filarial infection co-endemic with *L. loa* [55] and therefore a potential concern for cross-reactivity. One study evaluating an ELISA detecting IgG4 against *L. loa* adult worm antigen using sera from patients with only *M. perstans* infection and clearly identified as coming from *L. loa* non-endemic areas, found a high specificity (93.8%; 95%CI 67.7-99.7) [56] (Fig 4A) while Gobbi et al [54] found a specificity of 81.1% (95%CI 64.3-91.4%) when applying the IgG-detecting Ll-SXP-1-based RDT on the same type of samples (Fig 4C). No cross-reactivity was observed in the only single sample available from a patient with *Brugia* infection [54].

As expected, positivity was found with the pan-filarial ELISA based on antigens from *A. viteae* using sera from patients with *M. perstans* (35/37 positive) and *Brugia* (1/1 positive) infection from *L. loa* non-endemic areas [54]. When evaluated with sera from patients from *L. loa* non-endemic areas with other parasitoses, cross reactivity was found with *Strongyloides* (11/18 positive), *Ancylostoma* (3/5 positive), and *Schistosoma* (1/20 positive), for an estimated specificity of 68.1% (95%CI 52.7-80.5%) [54].

Only one study [45] evaluated an antigen-capture ELISA detecting the Ll-Bhp-1 antigen, finding a specificity of 100% on both a cohort of 34 sera from non-endemic controls, and a cohort of sera from patients with *Wuchereria bancrofti, Onchocerca volvulus*, and *M. perstans* from *L. loa* non-endemic areas.

When evaluating specificity using samples from individuals from *L. loa* endemic areas, specificity values were generally lower (Table 3, Fig 4 and S6 File). Since cross-reactivity with *M. perstans* and other parasitoses would seem minor for *L. loa*-specific antibody-detecting serology assays, these lower specificities could be either attributed to exposure/past infection, or reflect current infection not detected by the reference test, because a lower specificity could also be obtained when the assay has higher sensitivity compared to the reference test.

**Table 3:** Summary of specificity ranges of *L. loa*-specific antibody-detecting serology assays by subgroups. The table shows the minimum and maximum estimated specificity for each assay category, and the reference test against which the specificity was measured, when using sera from *L. loa* endemic areas. “[Pooled/single]” indicates whether the reported specificity was pooled from multiple comparisons or obtained from the results of a single comparison. Specificity against non-endemic control sera is not reported in the table since it was always 100%.

Assay	Minimum specificity		Maximum specificity	
	Reference test	Sp (95%CI) [pooled/single]	Reference test	Sp (95%CI) [pooled/single]
**Serology Ab-ELISA**				
**Mp-pos Loa-neg**	Microscopy, thick blood smear	10.3% (4.3-21.8) [single]	Composite: Microscopy mf concentration + PCR	28.9% (0-100) [pooled]
**Mp-neg Loa-neg**	Composite: Microscopy mf concentration + PCR	16.7% (14.0-20.0) [single]	Microscopy, thick blood smear	21.8% (14.0-32.2) [single]
**Loa-neg (Mp not specified)**	Composite: Microscopy mf concentration + PCR	26.0% (20.0-63.1) [pooled]	RAPLOA	49.1% (0.0-) [pooled]
**Serology Ab-RDT**				
**Mp-pos Loa-neg**	Composite: Microscopy mf concentration + PCR	57.7% (51.7-63.4) [pooled]	Microscopy, mf concentration	81.1 (64.3-91.4) [single]
**Mp-neg Loa-neg**	n.a	n.a.	Composite: Microscopy mf concentration + PCR	64.8% (59.9-69.4) [single]
**Loa-neg (Mp not specified)**	Composite: Microscopy mf concentration + PCR	71.4% (68.4-74.3) [single]	RAPLOA	72.9% (69.5-76.1) [single]

Mf = microfilariae. Mp = *Mansonalla perstans*. RDT = immunochromatographic rapid test (all based on recombinant Ll-SXP-1 antigen). Ab-ELISA = Antibody-detecting ELISA. Ab-RDT = Antibody-detecting RDT. N.a. = not applicable; not possible to provide a range since only one study is available the results of which are indicated arbitrarily in the “Maximum specificity” column.

#### Microscopy-based and molecular techniques.

As mentioned, for the purpose of analysis, microscopy-based and molecular assays were considered reference assays with perfect specificity. Nevertheless, we evaluated their specificity to highlight different aspects of these assays.

Specificity of microscopy-based techniques is operator dependent, since the recognition of mf at species level depends on the skills of the microscopist, the quality of slide preparation and of the microscope. Therefore, specificity in practice could be not always 100%. One study [49] estimated the specificity of thick blood smears and leukococoncentration using LCA, finding a pooled specificity of 99.7% (95%CI 99.2-100%) and 95.7% (95%CI 91.3-98.7), respectively (S6 File). These results confirm, at least in this study, the high reliability of *L. loa* mf identification by microscopy.

We also evaluated the specificity of molecular assays in the light of the consideration that lower specificity could also be obtained when the assay has higher sensitivity compared to the reference test. PCR-based techniques had a pooled specificity of 85.8% (95%CI 71.0-96.1; reference tests: microscopy of concentrated mf), with PCR results tending towards the upper range of specificity in studies carried out outside endemic areas (S6 File). The pooled specificity of LAMP was between 93.1% (95%CI 86.8-97.6; reference test microscopy of thick smears) and 94.4% (95%CI 90.3-97.6; reference test PCR) (S6 File).

### Quality assessment

Only studies from which sensitivity and specificity figures could be derived were evaluated for quality. Using an adapted version of the Newcastle – Ottawa quality assessment Scale, only three studies (3/27; 11.1%) could be classified as high quality (4–5 items with high quality over a total 6 items scored); most studies (18/27; 66.7%) had either a score of 2 or 3 (9/27; 33.3% studies for each score) (S4 File). In the “selection” domain, the majority (18/27; 66.7%) of studies were population-based carried-out in endemic areas. However, less than half (13/27; 48.1%) included microscopy examination of daytime microfilaraemia from a large volume of blood (either alone or in a composite standard) as the reference test. The same percentage considered potential sources of cross-reactivity/false positivity, and only one (1/27; 3.7%) study defined participants as uninfected with *L. loa* after exclusion of eyeworm and Calabar swelling history. In the “comparability” domain, 17/27 (63.0%) studies derived infected and uninfected individuals from comparable groups of individuals. Finally, no study openly stated whether the operators implementing the index tests were blinded to the results of the reference tests (“outcome” domain). Due to the high heterogeneity of the combinations reference-index tests and the fact that only 3 studies were of high quality, we opted for not performing an analysis stratified by quality.

Sources of bias deriving from the study designs and reporting shortcomings could be identified as selection bias (generally biasing results towards better performance) and verification bias (biasing results towards both directions). It must be also noted that incorporation bias (biasing results towards better performance) could have been introduced by the authors of this review when composite reference standards were purposively built with inclusion of the index test (e.g., a PCR being the index test was evaluated for sensitivity against a composite reference standard including positivity in microscopy and/or the same PCR).

## Discussion

The aim of this systematic review was to provide a landscape of the laboratory-based techniques available for the diagnosis of infection with *L. loa*, and their estimated sensitivity and specificity in individuals from endemic and non-endemic regions. We found a highly heterogeneous scenario, with even classical parasitological techniques deployed routinely in endemic (mostly thick blood smears) and non-endemic (mostly mf concentration techniques) areas not being applied in a standardized manner. At least for the former setting, this could be partly due to the final aim for which the technique was applied, for example whether it was applied for a prevalence assessment or for triaging only individuals with high *L. loa* mf burden. This heterogeneity of techniques was even more evident for molecular tests and serological assays, with no protocol/kit so far having reached commercial availability apart from the pan-filarial ELISA test used in Gobbi et al. [54], which uses *A. viteae* as source of antigens (Bordier Affinity Products, Crissier, Switzerland) and is therefore not *L. loa*-specific. This could be a further obstacle to the diagnosis of loiasis in the clinical context outside endemic areas in Europe, since the new European In Vitro Diagnostic Regulation (IVDR) [57] requires European diagnostic laboratories to evaluate the performance and accredit their in-house assays to issue diagnostic reports, encompassing the development and maintenance of detailed documentation of the entire test lifecycle (design, performance, risk management, clinical use, follow-up), if no commercial CE-IVDR marked test is available.

The significance of serology in endemic and non-endemic settings differs, since in endemic areas a very high percentage of individuals do have detectable antibodies against *L. loa*. The correlation of seropositivity and true active but amicrofilaraemic infection is unknown due to a missing gold-standard for diagnosing amicrofilaraemic infection. This was well remarked by Dieki et al [58], who evidenced how only some amicrofilaraemic individuals who were IgG4 positive on ELISA recognized *L. loa* antigens in Western Blot. This makes the usefulness of serology for screening questionable, in this setting. In the clinical setting, where the final goal is to identify patients with ongoing infection and treat them to reach parasitological cure, a common approach is to perform first screening with one or more techniques achieving high sensitivity, followed by a specific technique to confirm infection and guide treatment. The results of this review (Table 2 and Fig 3) suggest that serological ELISA assays or a molecular-based test could serve a screening purpose, with the advantage of ELISA over a molecular assay to likely allowing to achieve higher sensitivity for patients with amicrofilaraemic loiasis. The recombinant antigen-based Ll-SXP-1 RDT seems less fit for this purpose in the clinical setting, but results were very variable, probably also due to different cut offs used for positivity, between the few studies having applied this test, not allowing reliable conclusions. However, it needs to be highlighted that it has been shown that serological assays (ELISA and Ll-SXP-1 RDT) may be false negative in highly microfilaraemic individuals [51], and even Western Blot may be negative in some microfilaraemic individuals [58]. Thus, while serological assays may be used as a first screening test, a negative serological test cannot reliably exclude a microfilaraemic (or amicrofilaraemic) *L. loa* infection. Therefore, whatever serological assay is applied for screening, it always needs to be accompanied by a test for direct mf detection before initiation of an antiparasitic treatment with potential side effects in *L. loa*-infected individuals [24,59] when in the presence of *L. loa* epidemiological and/or clinical suggestive factors. Importantly, microscopy needs to be done on blood drawn at daytime (10 am-2 pm) [60] to provide a specific diagnosis of microfilaremia and mf counts to guide treatment. Indeed, while a linear correlation is described between mf count and PCR Ct value [51], it needs to be remembered that PCR Ct values are influenced by many factors, including the DNA target, blood volume, DNA extraction method and volume applied, PCR protocol, machine and others. Since at present thresholds for the risk of adverse events after treatment with rapidly-acting drugs against mf (diethylcarbamazine, ivermectin) are only established based on microscopy-based mf counts [61,62], also a positive PCR result for *L. loa* must be followed by conventional microscopy.

Two main methods are applied for the detection of circulating mf: thick blood smear, using microliters of blood, possibly from finger prick, and mf concentration techniques (leukoconcentration or filtration methods), performed with millilitres of venous blood. Compared to mf concentration techniques, a thick blood smear requires less laboratory equipment (e.g., no centrifuge), is less time-consuming, and is commonly deployed in endemic areas, especially when missing the detection of low-level microfilaraemia (not at risk of adverse events after treatment) is not critical for treatment safety considerations. The results of our review seem surprisingly pointing towards thick blood smear and mf concentration techniques having comparable sensitivity. One publication [48] compared the use of capillary and venous blood for the detection of mf by thick smears, reporting higher mf counts in capillary blood. However, whether a higher concentration of mf in capillary compared to venous blood can explain similar sensitivities of techniques using such large difference of blood volume is questionable, and difference in the populations and settings where the techniques were most frequently applied (thick blood smears in endemic areas and mf concentration techniques in non-endemic areas) could be at the basis of these results. Indeed, when considering the only two studies [49,50] comparing directly thick blood smears and mf concentration techniques using the same reference standard, the pooled sensitivity of mf concentration techniques was higher (99.9%, 95%CI 96.3-100%) than that of thick blood smears (79.9%, 95%CI 71.2-87.1%), suggesting that, when aiming at sensitivity of mf detection and when possible, applying a concentration technique would be preferable.

This study had several limitations. First, the search strategy was constructed with terms referring to the diagnosis of loiasis; therefore, publications applying a diagnostic technique for the diagnosis of infection with *L. loa* but not indexed with the terms selected for the search could have been missed. However, based on the knowledge of the authors, no known technique applied or investigated for the diagnosis of loiasis was evidently missed from the landscape analysis. Second, and most importantly, due to the high heterogeneity of the techniques used (and methods within each technique) as index and reference tests, and the study design applied to the majority of studies, we limited our analysis to estimating sensitivity and specificity of assays categories independently, and grouped by reference method. In addition, for the same reasons, we did not formally carry out a comparison between performance of assays by setting and origin of samples (endemic vs non-endemic area and samples obtained from continuously-exposed individuals vs travellers only temporarily exposed). However, as evidenced in forest plots presented in S5 and S6, results from samples obtained in studies carried out outside endemic areas, and on samples not obtained in endemic areas, were overall within the ranges obtained in endemic areas. When a trend difference was identified and highlighted in the relevant results section, this was towards higher sensitivity or specificity obtained in studies outside endemic areas, but this could in part be due to the predominant case-control diagnostic accuracy design of these studies. While this does not provide a conclusive head-to-head comparative analysis of different techniques, this analysis provides a comprehensive picture of current data available on the performance of diagnostic assays deployed or developed and tested for the diagnosis of loiasis. Third, the quality of studies was overall low, and different sources of bias (the overall direction of which is difficult to judge) could have influenced our results. Also notably, for the clinical setting, there is a lack of studies evaluating different diagnostic techniques/assays on a prospective (or even retrospective) cohort of samples obtained from patients attended in the actual setting where the tests/assays would be deployed, that is, consecutive patients with epidemiological and/or clinical suspicion of infection with *L. loa*.

## Conclusions

Loiasis is a largely neglected filarial infection poorly known outside endemic areas but even underappreciated in endemic countries. Due to the potentially severe consequences of misdiagnosis, its correct identification and circulating mf load quantification are paramount to guide treatment. We carried out a systematic review of the literature and provided a landscape of diagnostic methods and assays for the diagnosis of loiasis and a comprehensive picture of current data available on their performance. Our results show high heterogeneity of the methods and assays applied and/or developed for the diagnosis of this filarial infection. Even in the case of classic parasitological techniques there was no standardization, and currently no molecular or serological assays specific for the diagnosis of loiasis is commercially available. Thus, a robust comparison of the performance of each available technique was not possible. However, the results of this review point towards ELISA serology and possibly PCR being appropriate for screening in the clinical context, followed by microscopy in case of positivity and even in case of negativity but presence of epidemiological or clinical factors anyway suggestive of loiasis. Large, multicentric studies on cohort of samples mirroring the population of patients on which diagnostics for loiasis would be deployed are needed to support the development of evidence-based diagnostic algorithms for this infection.

## Supporting information

S1 File Preferred Reporting Items for Systematic Reviews and Meta-Analyses of Diagnostic Test Accuracy studies (PRISMA-DTA). Adapted From: McInnes MDF, Moher D, Thombs BD, McGrath TA, Bossuyt PM, The PRISMA-DTA Group (2018). Preferred Reporting Items for a Systematic Review and Meta-analysis of Diagnostic Test Accuracy Studies: The PRISMA-DTA Statement. JAMA. 2018 Jan 23;319(4):388–396. https://doi.org/10.1001/jama.2017.19163.
(DOCX)

S2 File Literature search strategy. (DOCX)

S3 File Newcastle-Ottawa Quality Assessment Scale. (DOCX)

S4 File Raw data and quality assessment. (XLSX)

S5 File Sensitivity of assays. (PDF)

S6 File Specificity of assays. (PDF)
