# Supplementary material for: Laboratory diagnosis of loiasis to support individual patient management: A systematic review
Source: PLoS Negl Trop Dis. 2026 Jul 13;20(7):e0014460. doi: 10.1371/journal.pntd.0014460 (PMC13379093; doi:10.1371/journal.pntd.0014460)
Supplement: S2 File — (DOCX) [file pntd.0014460.s002.docx]

**Search strategy**

**MEDLINE (Pubmed) (1946 to 26 March 2025)**

((("Loa"[MeSH Terms] OR "Loiasis"[MeSH Terms] OR ("Loa"[Title/Abstract] OR "Loa loa"[Title/Abstract] OR "loias*"[Title/Abstract] OR "loa loa infection*"[Title/Abstract] OR "loa loa filarias*"[Title/Abstract])) OR "loase"[Title/Abstract] OR "loaose"[Title/Abstract]) OR ("Calabar"[Title/Abstract] OR "African eye worm"[Title/Abstract] OR "filaria lacrimalis"[Title/Abstract])) AND ((("Diagnosis"[MeSH Terms:noexp] OR "diagno*"[Title/Abstract]) OR ("Serology"[MeSH Terms] OR "Serologic Tests"[MeSH Terms] OR "Antibodies"[MeSH Terms] OR "Rapid Diagnostic Tests"[MeSH Terms] OR "Nucleic Acid Amplification Techniques"[MeSH Terms] OR "Polymerase Chain Reaction"[MeSH Terms] OR "Antigens"[MeSH Terms] OR ("serologic test*"[Title/Abstract] OR "serological test*"[Title/Abstract] OR "serodiagnos*"[Title/Abstract] OR "Serology"[Title/Abstract] OR "seroassay"[Title/Abstract] OR "antibod*"[Title/Abstract] OR "serodiagnos*"[Title/Abstract] OR "rapid test*"[Title/Abstract] OR "molecular*"[Title/Abstract] OR "nucleic acid amplification*"[Title/Abstract] OR "polymerase chain reaction*"[Title/Abstract] OR "PCR"[Title/Abstract] OR "Anchored PCR"[Title/Abstract] OR "Anchored Polymerase Chain Reaction"[Title/Abstract] OR "Inverse PCR"[Title/Abstract] OR "Inverse Polymerase Chain Reaction"[Title/Abstract] OR "Nested Polymerase Chain Reaction"[Title/Abstract] OR "Nested PCR"[Title/Abstract] OR "LAMP assay"[Title/Abstract] OR "LAMP"[Title/Abstract] OR "isothermal amplification"[Title/Abstract] OR "antigen*"[Title/Abstract] OR "microfilarial count"[Title/Abstract] OR "microscopy"[Title/Abstract] OR "blood film"[Title/Abstract] OR "blood smear"[Title/Abstract] OR "leucoconcentration"[Title/Abstract] OR "filtration"[Title/Abstract] OR "LoaScope"[Title/Abstract]))) OR ("ELISA "[Title/Abstract] OR "Immunoassay"[Title/Abstract]))

**Embase (1974 to 26 March 2025)**
#1 'loiasis'/exp/mj

#2 'loa loa' OR 'loiasis' OR 'loa loa infection*' OR 'loa loa filarias*' OR loase OR loaose OR calabar OR 'african eye worm' OR 'filaria lacrimalis':ti,ab

#3 #1 or #2

#4 'antibody'/exp/mj OR 'rapid test'/exp/mj OR 'nucleic acid amplification techniques'/exp/mj OR 'polymerase chain reaction'/exp/mj OR'antigen'/exp/mj OR 'serology'/exp/mj

#5  'serologic test*' OR 'serological test*' OR serology OR seroassay OR antibod* OR serodiagnos*
     OR 'rapid test*' OR molecular* OR 'nucleic acid amplification*' OR 'polymerase chain reaction*'
     OR pcr OR 'anchored pcr' OR 'anchored polymerase chain reaction' OR 'inverse pcr' OR 'inverse
     polymerase chain reaction' OR 'nested polymerase chain reaction' OR 'nested pcr' OR 'lamp assay'
     OR lamp OR 'isothermal amplification' OR antigen* OR micrifialaremia OR 'microfilarial count' OR
     microscopy OR 'blood film' OR 'blood smear' OR leucoconcentration OR filtration OR 'loascope' OR ELISA OR Immunoassay:ti,ab
#6 .  'diagnosis'/exp/mj OR ('diagnosis':ti,ab)

#7 #5 OR #6

#8 #3 AND #7

**Cochrane Central Register of Controlled Trials (CENTRAL; 2025, Issue 3) in the Cochrane Library (searched 26 March 2025)**

#1 MeSH descriptor: [Loiasis] explode all trees

#2 ('loa loa' OR 'loiasis' OR 'loa loa infection*' OR 'loa loa filarias*'):ti,ab,kw

#3 #1 OR #2

#4 diagnosis

#5 #3 AND #4
