# Supplementary material for: Laboratory diagnosis of loiasis to support individual patient management: A systematic review
Source: PLoS Negl Trop Dis. 2026 Jul 13;20(7):e0014460. doi: 10.1371/journal.pntd.0014460 (PMC13379093; doi:10.1371/journal.pntd.0014460)
Supplement: S3 File — (DOCX) [file pntd.0014460.s003.docx]

**Adapted version of the Newcastle-Ottawa Quality Assessment Scale used to rank the quality of the studies included in the meta-analysis.**

| **Domains** | **Items** |
| --- | --- |
| **Selection** | 1. Study design and representativeness of the cases 2. Population-based study design in endemic area 🟏 3. Other study designs |
|  | 1. Reference diagnostic test 2. Based on concentration technique of large volume of blood taken around noon🟏 3. Based on other microscopy techniques or NAAT or composite reference standard or LCA (not including technique in a.) or not meeting/uncertain description of point a. 4. RAPLOA |
|  | 1. Uninfected patients’ classification description 2. Uninfected people from endemic area (history of eyeworm and Calabar excluded)🟏 3. Uninfected people from endemic area (history of eyeworm and Calabar not excluded)   c. Other controls categories |
|  | 1. Evaluation of cross-reactivity   a. Relevant sources of potential cross-reactivity considered🟏  b. Not explored or not clearly indicated |
| **Comparability** | 1. Infected and non-infected individuals are from comparable patient groups   a. Yes🟏  b. No |
| **Outcome** | 1. Blind regarding results of the reference assay by personnel implementing the index test   a. Yes 🟏  b. No/not stated |

**Low quality: studies awarded 1 to 3 stars; high quality: studies awarded 4 or 5 stars; very high quality: studies awarded 6 stars.**
