# Supplementary material for: Laboratory diagnosis of loiasis to support individual patient management: A systematic review
Source: PLoS Negl Trop Dis. 2026 Jul 13;20(7):e0014460. doi: 10.1371/journal.pntd.0014460 (PMC13379093; doi:10.1371/journal.pntd.0014460)

1.SENSITIVITY OF MICROSCOPY THICK SMEAR.

1.1 vs reference of composite microscopy

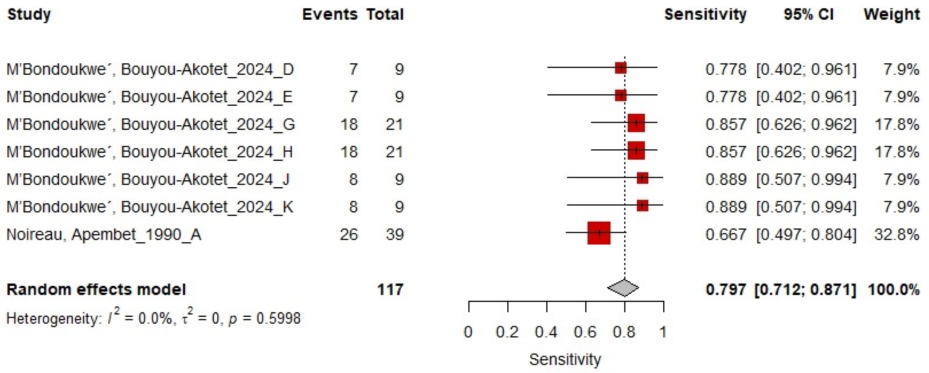

1.2. vs reference of microscopy concentration

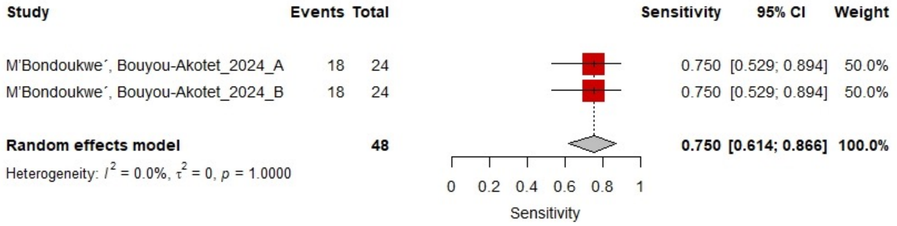

1.3. vs reference of composite microscopy thick smear + PCR

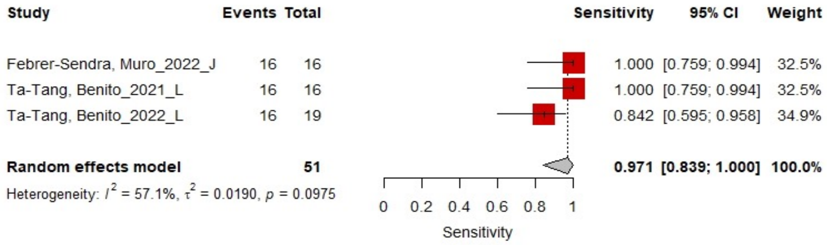

1.4. vs reference of PCR

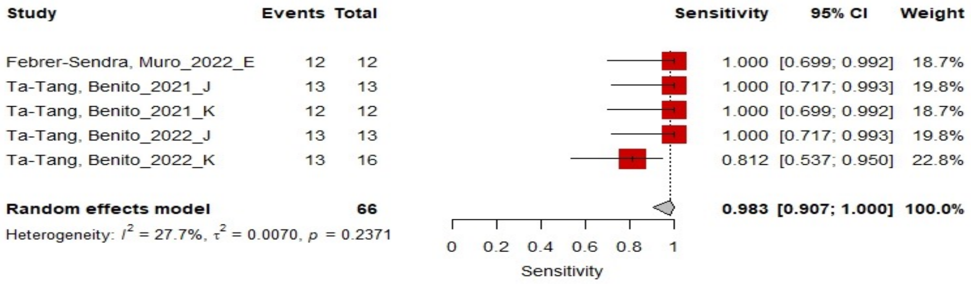

2. SENSITIVITY OF MICROSCOPY CONCENTRATION TECHNIQUES

* = studies performed on samples not collected in endemic areas

2.1. vs reference of composite microscopy concentration + PCR

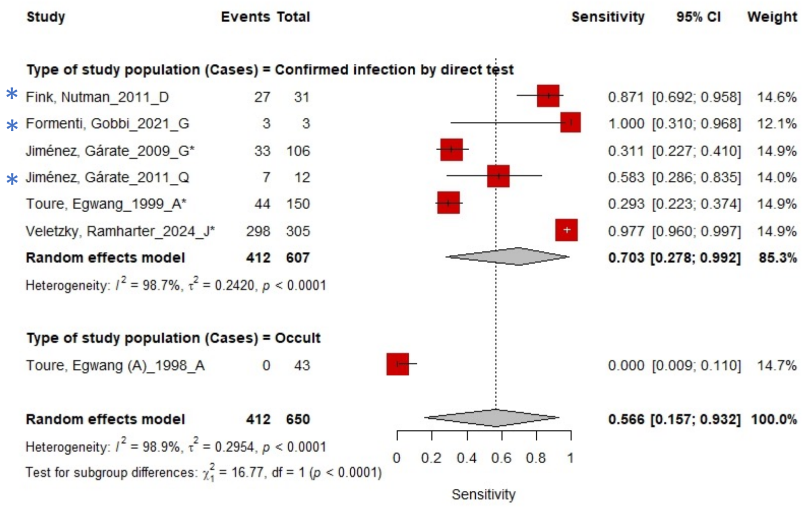

2.2. vs reference of PCR

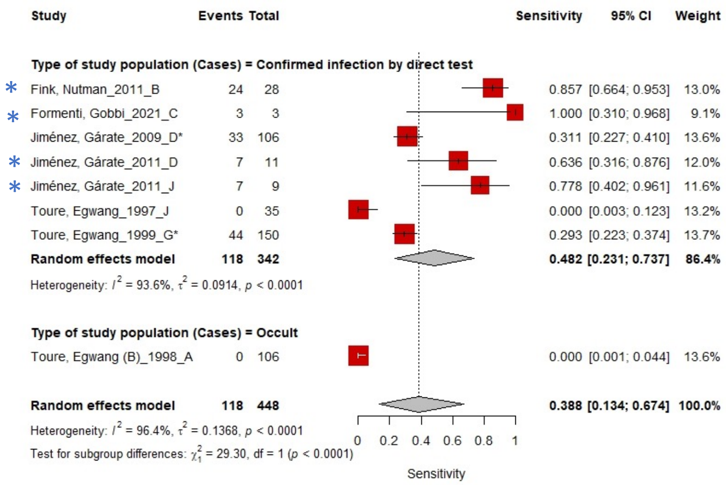

2.3. vs reference of composite microscopy

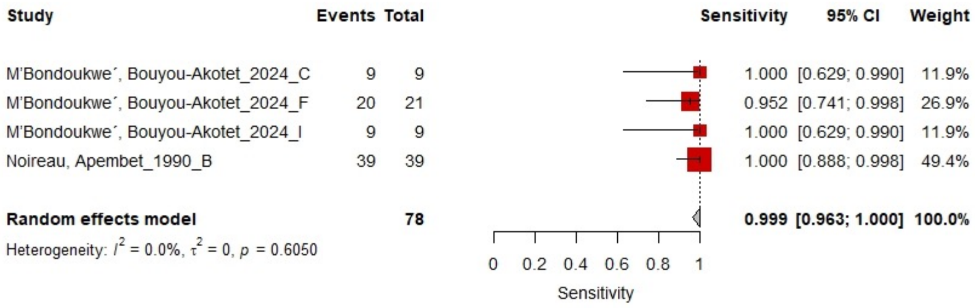

3. SENSITIVITY OF MOLECULAR TECHNIQUES_PCR

* = studies performed on samples not collected in endemic areas

3.1. vs reference of composite eyeworm obs. + microscopy_concentration

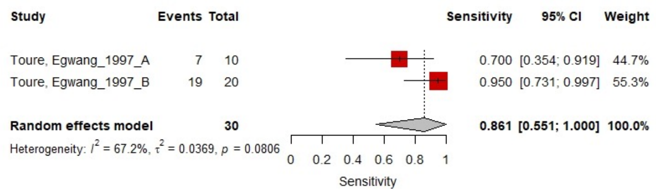

3.2. vs reference of composite microscopy_thick smear + PCR

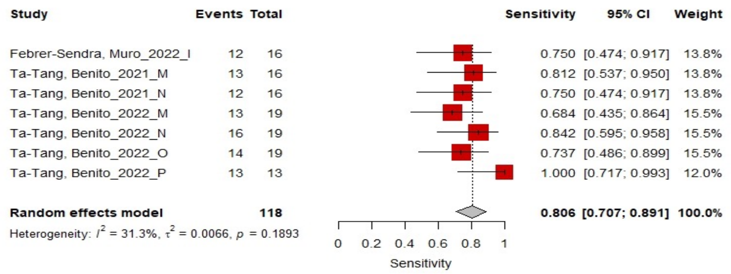

3.3. vs reference of microscopy thick smear

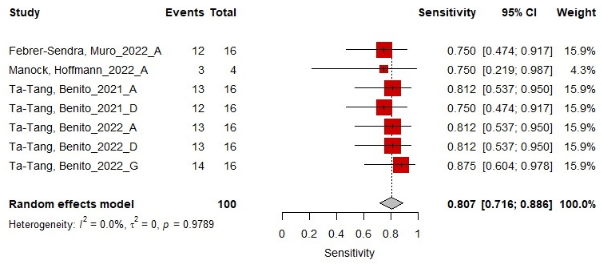

3.4. vs reference of composite microscopy_concentration + PCR

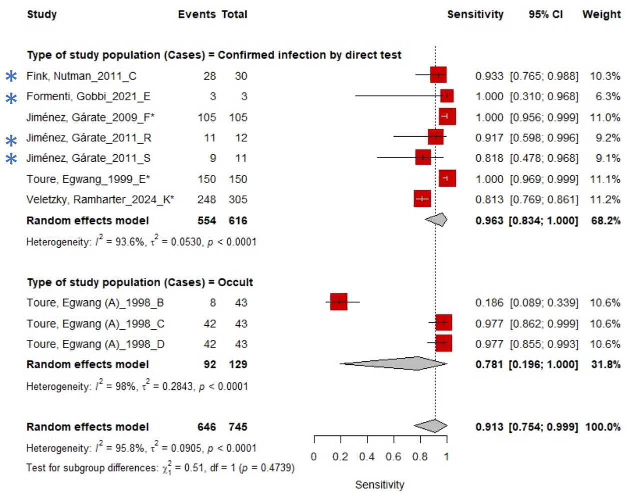

3.5. vs reference of RAPLOA

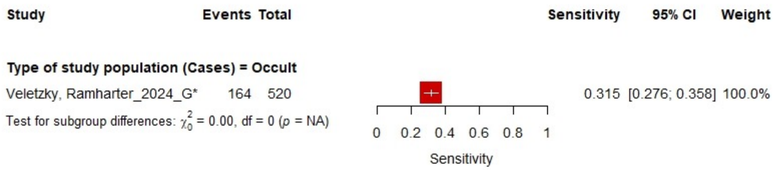

3.6. vs reference of microscopy conc.

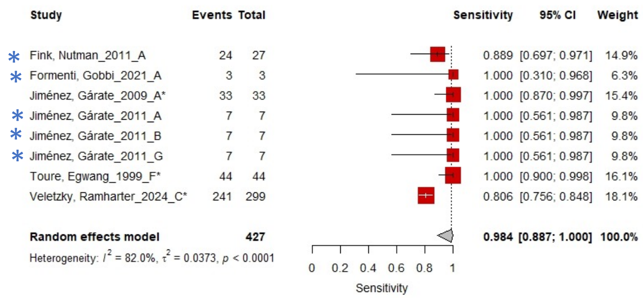

4. SENSITIVITY OF molecular techniques_LAMP

4.1. vs reference of composite microscopy_thick smear + PCR

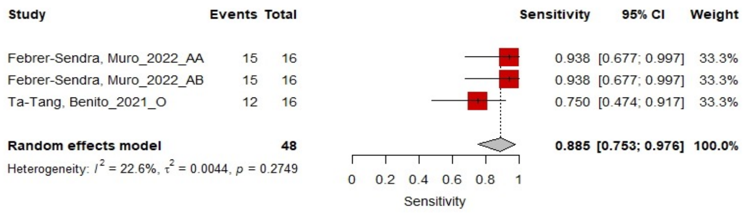

4.2. vs reference of PCR

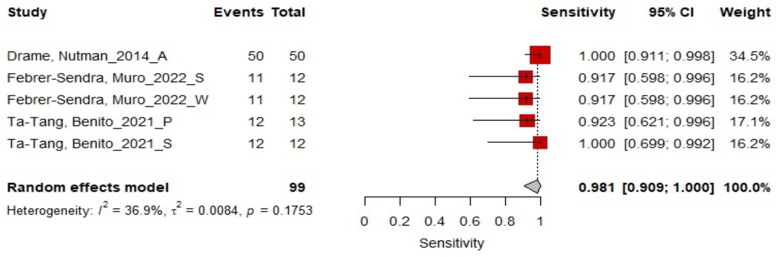

4.3. vs reference of microscopy_thick smear

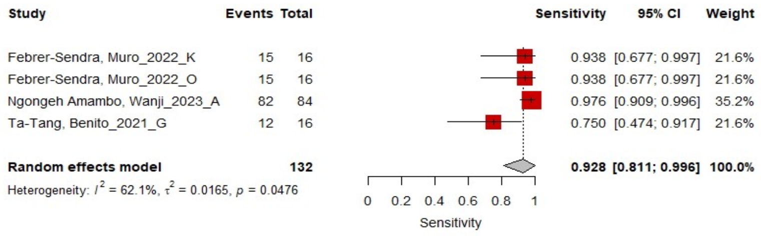

5. SENSITIVITY OF SEROLOGY TECHNIQUES_ELISA

* = studies performed on samples not collected in endemic areas

5.1. vs reference of composite microscopy_concentration + PCR

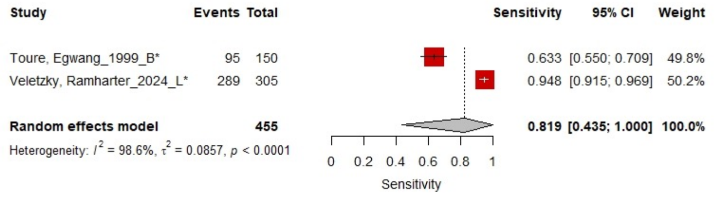

5.2. vs reference of RAPLOA

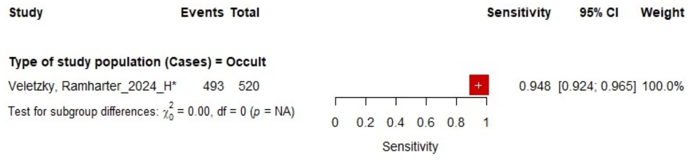

5.3. vs reference of composite eyeworm and microscopy

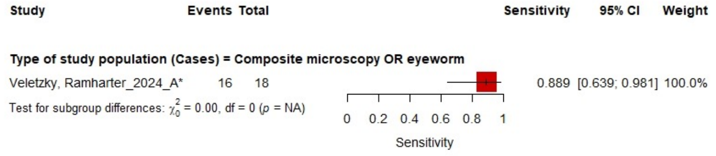

5.4. vs reference of microscopy concentration

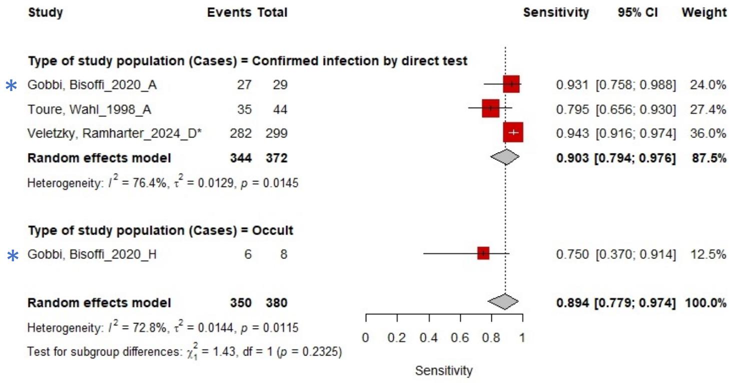

5.5. vs reference of microscopy thick smear

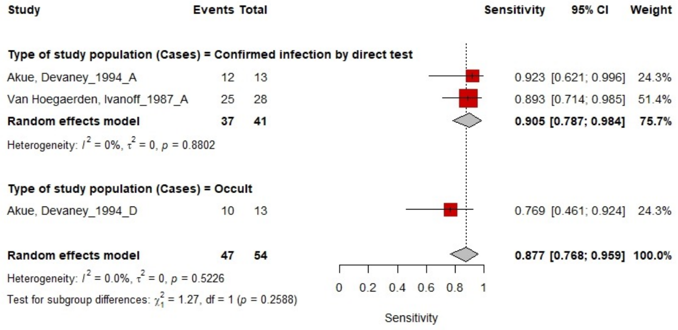

5.6. vs reference of composite eyeworm and microscopy conc.

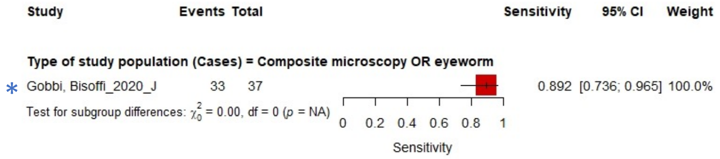

6 SENSITIVITY OF SEROLOGY TECHNIQUES_RDT

* = studies performed on samples not collected in endemic areas

6.1. vs reference of microscopy thick smear

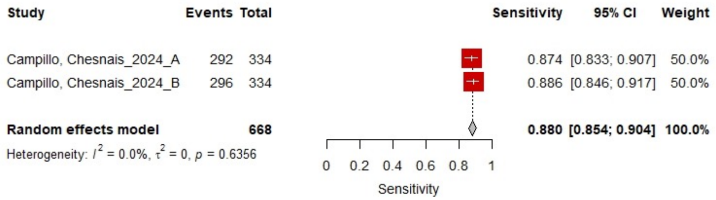

6.2. vs reference of RAPLOA

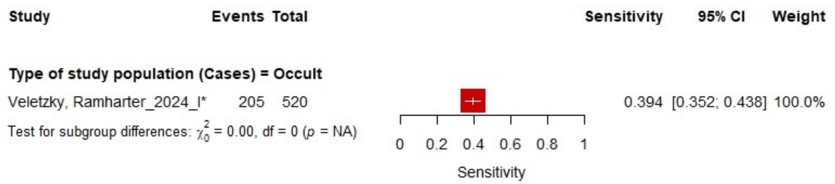

6.3. vs reference of microscopy concentration

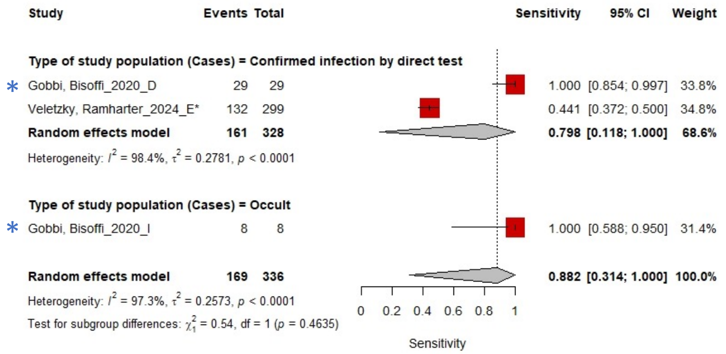

6.4. vs reference of composite microscopy conc. + PCR

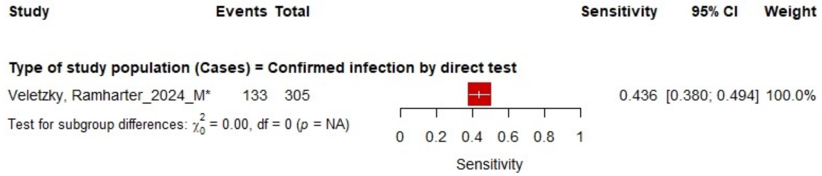

Supplement: S5 File — (PDF) [file pntd.0014460.s005.pdf]
