# Supplementary material for: Laboratory diagnosis of loiasis to support individual patient management: A systematic review
Source: PLoS Negl Trop Dis. 2026 Jul 13;20(7):e0014460. doi: 10.1371/journal.pntd.0014460 (PMC13379093; doi:10.1371/journal.pntd.0014460)

1. SPECIFICITY OF MICROSCOPY THICK SMEAR.

1.1 vs reference of composite microscopy

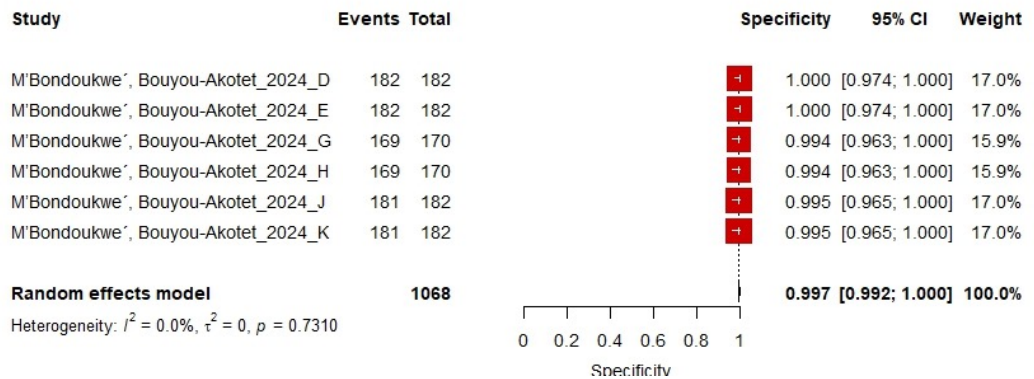

2. SPECIFICITY OF MICROSCOPY CONCENTRATION TECHNIQUES

2.1 vs reference of composite microscopy

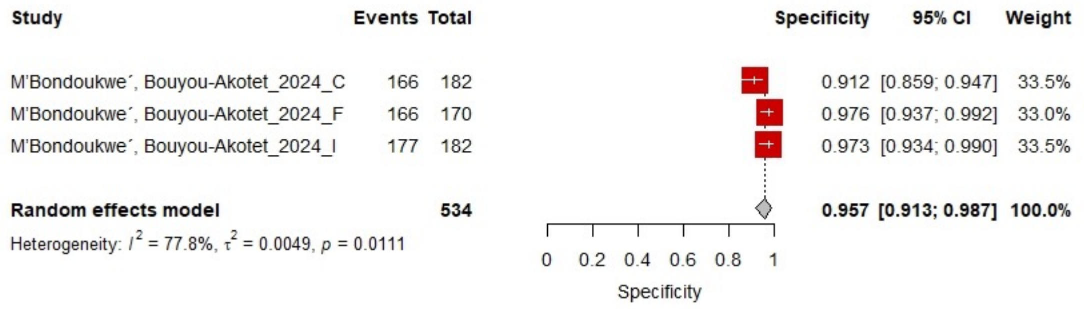

3. SPECIFICITY OF SEROLOGY_ELISA

* = studies performed on samples not collected in endemic areas

3.1 vs reference of RAPLOA

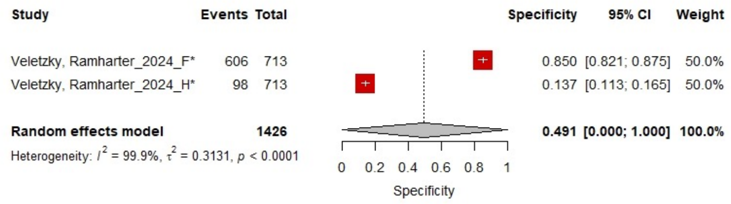

3.2 vs reference of composite eyeworm + microscopy

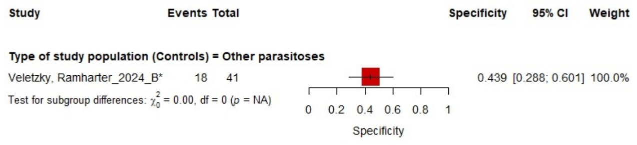

3.3 vs reference of microscopy concentration

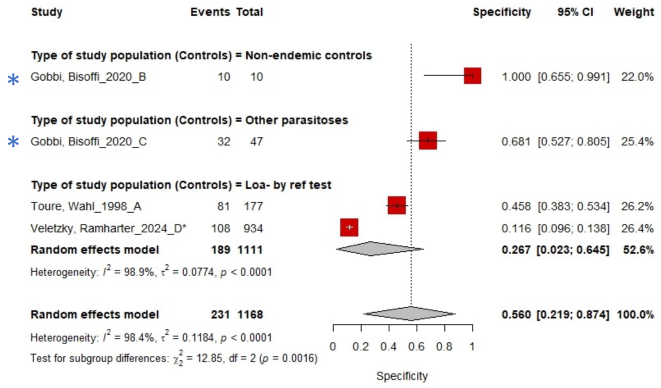

3.4 vs reference of microscopy thick smear

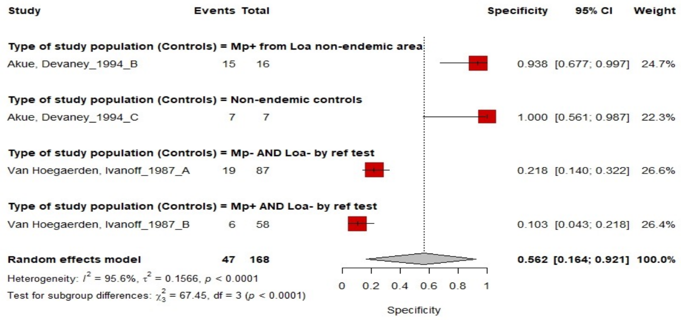

3.5 vs reference of composte microscopy conc. + PCR

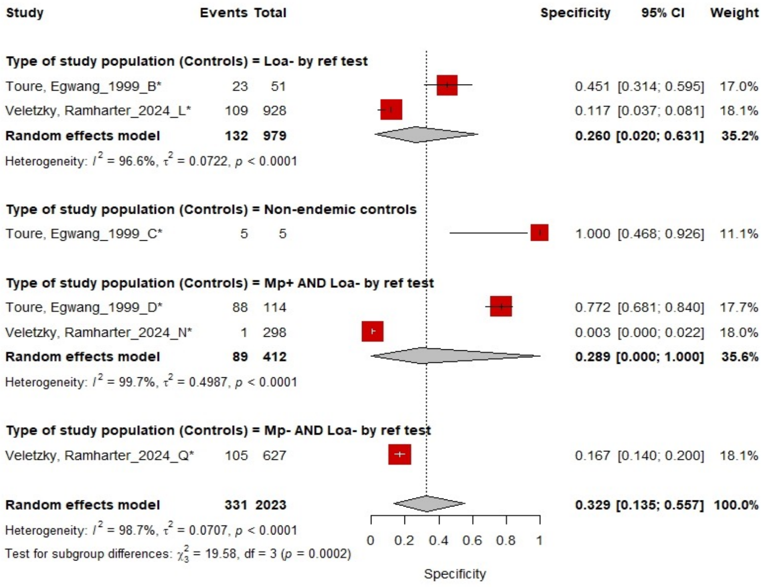

4. SPECIFICITY OF SEROLOGY_RDT

* = studies performed on samples not collected in endemic areas

4.1 vs reference of composite microscopy conc. + PCR

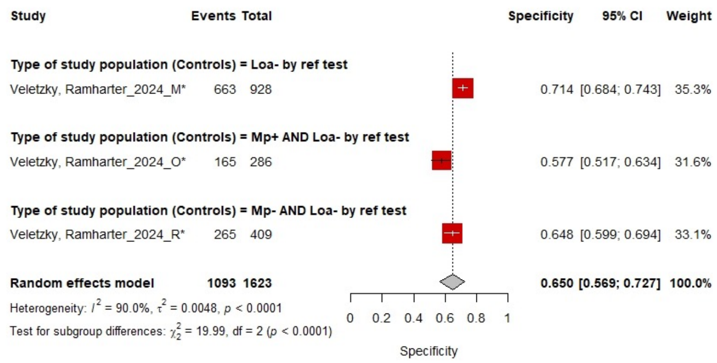

4.2 vs reference of microscopy concentration

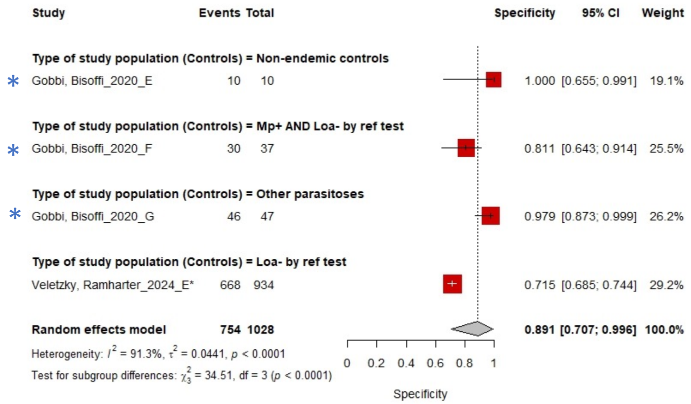

4.3 vs reference of RAPLOA

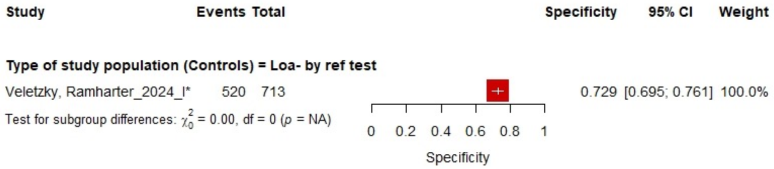

5. SPECIFICITY OF MOLECULAR_PCR (*considered 100% specific a-priori; low Sp interpretable as higher Se)*

* = studies performed on samples not collected in endemic areas

5.1 vs reference of microcopy concentration

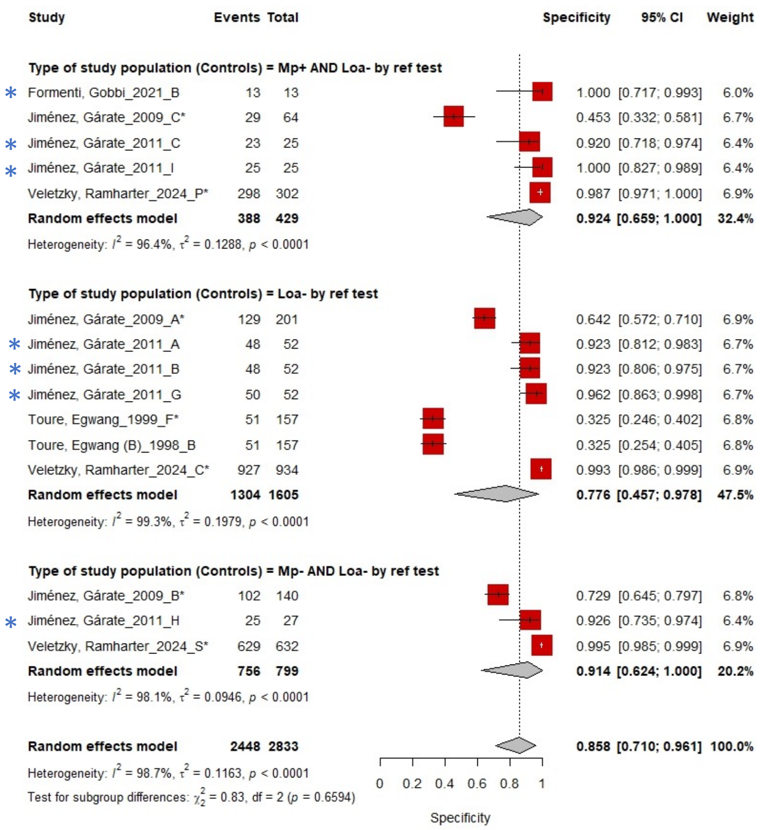

6. SPECIFICITY OF molecular_LAMP *(considered 100% specific a-priori; low Sp interpretable as higher Se)*

6.1 vs reference of microcopy thick smear

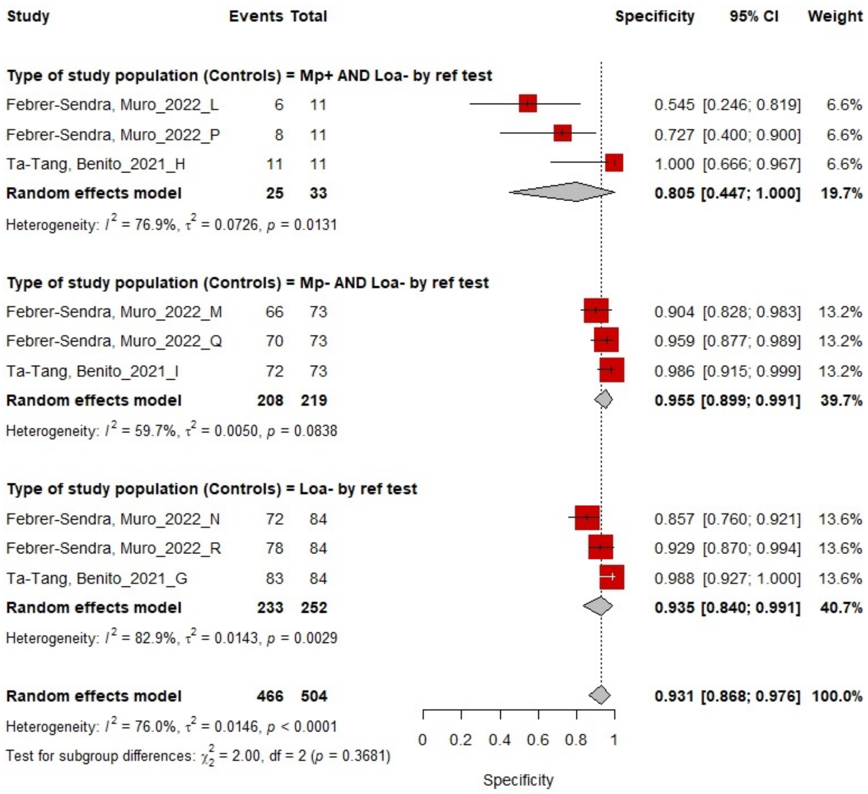

6.2 vs reference of PCR

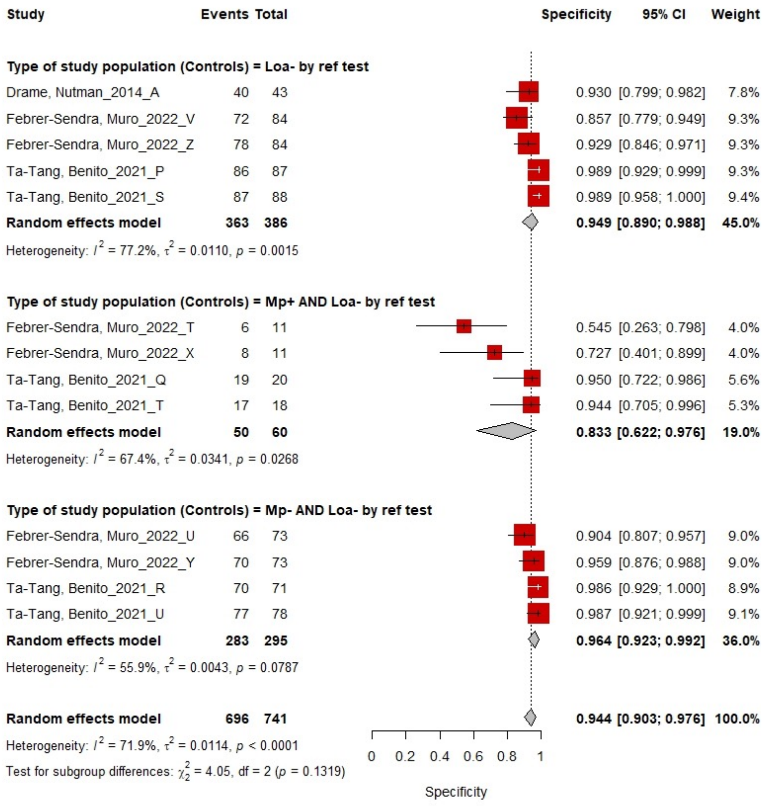

Supplement: S6 File — (PDF) [file pntd.0014460.s006.pdf]
