# Supplementary material for: Laboratory diagnosis of loiasis to support individual patient management: A systematic review
Source: PLoS Negl Trop Dis. 2026 Jul 13;20(7):e0014460. doi: 10.1371/journal.pntd.0014460 (PMC13379093; doi:10.1371/journal.pntd.0014460)
Supplement: Attachment — Submitted filename: Feedback and replies.docx [file pntd.0014460.s008.docx]

**NOTE FROM THE AUTHORS: we thank the reviewers for the time and attention dedicated to our work. The amendments made upon their remarks improved the accuracy, clarity and completeness of the paper. Please do note that we changed the continuous line numbers layout with line numbering starting at each page, due to the fact that in the R1 version with track changes there were unexplained line numbers missing. Therefore, when indicating where amendments were done in the text, in the replies below, we made reference to page (pp) and lines within that page.**

**Journal Requirements**:

Please amend your detailed Financial Disclosure statement. This is published with the article. It must therefore be completed in full sentences and contain the exact wording you wish to be published.
1) State the initials, alongside each funding source, of each author to receive each grant. For example: "This work was supported by the National Institutes of Health (####### to AM; ###### to CJ) and the National Science Foundation (###### to AM)."

**REPLY: the funding statement was amended as per instructions. Please note that for the Italian Ministry of Health, the way the funding statement is written is correct as it is, since this funding in not assigned to a person, but to the institution.**

2) State what role the funders took in the study. If the funders had no role in your study, please state: "The funders had no role in study design, data collection and analysis, decision to publish, or preparation of the manuscript.". If you did not receive any funding for this study, please simply state: u201cThe authors received no specific funding for this work.

**REPLY: the sentence about the role of funders was amended as per instructions.**

**Reviewers' Comments:**

**Methods**
Reviewer #1: the objective is clearly articulated, the study design and statistical analysis used support the conclusion.

**REPLY: we thank the reviewer for appreciating our work.**

Reviewer #2: Loiasis is an endemic parasitic disease in Central and West Africa that is underestimated, despite significant associated mortality and morbidity in endemic areas. This disease has come to attract attention in ‘Northern’ countries due to the diagnosis of imported cases in non-endemic areas.
This condition remains poorly understood and difficult to diagnose, with available diagnostic tools offering variable performance. In this manuscript, the authors have conducted a review of the literature on the various diagnostic techniques for loiasis in order to better understand the different tools available and their analytical performance. The short-term objective is to develop guidelines for the individual management of loiasis. This work is of interest to the scientific community given the lack of knowledge about loiasis and the methods for diagnosing it. The authors’ objectives are clear, the methodology is clearly explained, and the tables and graphs are useful and easy to understand, and the manuscript is well written.
However, certain methodological aspects still need to be developed and some results need to be revised in order to clarify this work.

**REPLY: we thank the reviewer for appreciating our work.**

Major revision:
1. Line 127: As stated by the authors, loiasis can present in various clinical and/or biological forms in infected patients. The aim of this study is to analyse all the diagnostic techniques used to detect Loa loa infection. However, the authors did not specify in the methodology what the definitions of Loa loa infection were in the various included studies (conjunctival passage of an adult, microfilaremia, positive serology +/- clinical symptoms +/- eosinophilia). Details of the various definitions used would provide a better understanding of the patient groups studied and put the results of the analytical performance of the different techniques into perspective.

**REPLY: thanks for this observation. We have checked the missing formation highlighted by the reviewer, however the actual details of what was considered “*Loa loa* infection” for our study were detailed in the following sentences: “*The target population was composed by individuals of all ages and both sexes, where infection with L. loa was assessed by microscopy or molecular analysis or “eyeworm” either witnessed by health personnel or identified as described by the WHO guidelines for rapid assessment of Loa loa (RAPLOA [28]). Eligible reference tests were direct microscopy-based or molecular assay or “eyeworm” identification as described above, or composites of these methods, or latent class analysis (LCA) encompassing these methods. Index tests were defined as any laboratory-based assay for the diagnosis of loiasis. Studies or groups of individuals within a study for which the diagnosis was based on clinical signs/symptoms or methods other than those detailed above were excluded, with the exception of uninfected controls who had never travelled/resided in L. loa endemic areas, who were considered bona fide uninfected*”. The results of this methods are presented as aggregated data in table 1, and per each paper in the raw data file (“*The database containing the complete list of included publications, data extraction and quality assessment is available as S4 File*.)”. We are therefore unsure what data was missing, and we are happy to make the needed amendments upon further clarification.**

2. Line 176-186: The clinical and laboratory presentation of loiasis can vary depending on the patient population ([https://doi.org/10.1093/infdis/163.6.1318](https://urldefense.proofpoint.com/v2/url?u=https-3A__doi.org_10.1093_infdis_163.6.1318&d=DwQFaQ&c=68L3JBqqiV6xi5WaZK0Zh1EsHqZczF0YL8bbEH5V5e8&r=CWP4W8Hgo_wBurBCgwLhzb5Pf-xLVeNBb6TMAJkEN_mpINiTkvz4G65-WNaM7IVD&m=Y8-7XxPtPltIcPsTsFHFBWF5GXgoWnFzZhJifC9wrOgnNjy7nplWsQTVuatI3G4U&s=s7PtHKT1ixig4ksYvqa_KpOKCf2nYYPdUYeLSp84xaw&e=)), as recently reviewed ([https://doi.org/10.1016/j.idnow.2025.105053](https://urldefense.proofpoint.com/v2/url?u=https-3A__doi.org_10.1016_j.idnow.2025.105053&d=DwQFaQ&c=68L3JBqqiV6xi5WaZK0Zh1EsHqZczF0YL8bbEH5V5e8&r=CWP4W8Hgo_wBurBCgwLhzb5Pf-xLVeNBb6TMAJkEN_mpINiTkvz4G65-WNaM7IVD&m=Y8-7XxPtPltIcPsTsFHFBWF5GXgoWnFzZhJifC9wrOgnNjy7nplWsQTVuatI3G4U&s=VO5MLszITdXkOJVBJ6YI13kNI2Dm4mNhoHBTyTQQbQQ&e=)). With a view to drafting future guidelines for the individual management of patients with loiasis, it is important to take into account the type of population, as the analytical performance of diagnostic tools may vary. Where possible, it would be useful to stratify analytical performance according to the study area: endemic (population continuously exposed and immunologically tolerant) and non-endemic (travellers with occasional exposure and low parasite load).

**REPLY: we thank the reviewer for this observation. We actually considered the suggested analysis when we were designed the analysis plan. However, only six studies were conducted outside endemic areas with samples from individuals not obtained in endemic areas (three studies conducted outside endemic areas were actually analyzing samples obtained in endemic areas, as reported in the raw data file), and in these six studies, it was not possible to extract data separately for migrants (continuously exposed in endemic areas) and travelers (just exposed for a brief period). In addition, when looking at the comparisons of assays performed in the six studies performed outside endemic areas on samples obtained outside endemic areas, they were comparing different assays, and they were making up only one or two of the studies, assessing the same broad category comparison, made in endemic and non-endemic settings. For all these reasons we deemed not appropriate to carry out this sub-analysis formally. However, we agree with the reviewer that highlighting and discussing this aspect is important; therefore, we have made the following amendments to the paper: 1) we have amended the S5 and S6 files by indicating with an asterisk the papers who provide data from samples obtained outside endemic areas, so that the reader can clearly see where the results place in comparison with those of studies carried out on samples obtained in endemic areas and we have explicitly made a reference to this in the results (pp10 line 28-30 and following pp11 line 1-4; pp14 line 8-14); 2) we have added a sentence pointing out this distinction in the results when a trend difference could be identified (pp12 line 17-19 and pp16 line 28-29); 3) we have added a discussion point in the discussion (pp20 line 7-14).**

**Results**
Reviewer #1: data are presented correctly

**REPLY: thank you, we are pleased that the way the data were presented resulted clear**

Reviewer #2: Major revision:
3. Line 220-301 and table 1: This paragraph is difficult to understand because there are numerous discrepancies between the figures given in the text and those in Table 1. There are also inconsistencies within Table 1 itself.

**REPLY: We thank the reviewer for the double checking of these sums. We have re-checked and cross-checked the calculations prompted by this input, and indeed we found some discrepancies of a couple of papers that were erroneously not updated in the table but only in the text calculations. We are sorry about this. We have amended the figures in the Table and we have also corrected a typo in pp9 line 14 (where we erroneously wrote 19 instead of 29). We have also amended slightly the sentence in pp8 line 25, to make understandable why the relative rows on Table 1 did not make up 91, and we have inserted a sentence summarizing from the beginning the total papers including a thick smear and an mf concentration technique (pp8 line 26-27), so that the denominators in the text and in the Table can be easier to follow and reconcile. The same was done for pp9 line 15-16 and line 26 to more easily reconcile the counts referring to molecular and seroassays. Please also note that the sums in the cells “Diagnostic methods” do not sum up to the total papers indicated in the Table’s columns since, as indicated by the symbol § in the caption, “*Single studies might have included more than one diagnostic method, therefore sums may exceed 100%*”. We also amended the titles and caption of Figure 2 to make all figures reconciled. Finally, as the consequence of the kind and correct remark from Reviewer #1 that one Western Blot from one of the papers included in the landscape analysis was not cited among the serological techniques used, we have amended all relevant figures to include this data.**

Line 227: microscopy was applied in 91/99 in the text but calculated as 96 in the table..?

**REPLY: We have amended the sentence in the text as follows: “At least one microscopy technique for the identification of mf was applied in the majority of studies (91/99, 91.9%)”. As noted in the table (§) and caption “*Single studies might have included more than one diagnostic method”.***

Line 229: “73%” I don't understand where that figure comes from?

**REPLY: this figure (as well as the one provided a few lines below for leukoconcentration in the non-endemic setting) referred to the total number of microscopy-based techniques applied in the papers in each setting, and the proportion of which were thick blood smears. However, we realized that this information was redundant, not particularly more informative than what already presented, and cumbersome to explain, therefore we erased the figures in the parentheses altogether.**

Line 237: leukoconcentration “32/35” in the text but estimated at 31 in the table.

**REPLY: We are unsure about the “31” figure the reviewer refers to. In table 1 all mf concentration methods were reported (26 + 9 =35), while the details of what mf concentration technique was applied is only detailed in the text.**

Line 244: Loascope at “5/99” but written at 7/99 in the table?

**REPLY: numbers were amended and reconciled between text and table. Thanks again for this cross-checking.**

Line 247 and 257: Discrepancies between text and table.

**REPLY: numbers were amended and reconciled between text and table. Thanks again for this cross-checking.**

4. “Performances assessment: sensitivity” section: where possible, presenting the accuracy of diagnostic techniques according to the study area (endemic/non-endemic) would help to put the results presented into context.

**REPLY: We thank the reviewer for the double checking of these sums. We have re-checked and cross-checked the calculations prompted by this input, and indeed we found some discrepancies of a couple of papers that were erroneously not updated in the table but only in the text calculations. We are sorry about this. We have amended the figures in the Table and we have also corrected a typo in pp9 line 14 (where we erroneously wrote 19 instead of 29). We have also amended slightly the sentence in pp8 line 25, to make understandable why the relative rows on Table 1 did not make up 91, and we have inserted a sentence summarizing from the beginning the total papers including a thick smear and an mf concentration technique (pp8 line 26-27), so that the denominators in the text and in the Table can be easier to follow and reconcile. The same was done for pp9 line 15-16 and line 26 to more easily reconcile the counts referring to molecular and seroassays. Please also note that the sums in the cells “Diagnostic methods” do not sum up to the total papers indicated in the Table’s columns since, as indicated by the symbol § in the caption, “*Single studies might have included more than one diagnostic method, therefore sums may exceed 100%*”. We also amended the titles and caption of Figure 2 to make all figures reconciled. Finally, as the consequence of the kind and correct remark from Reviewer #1 that one Western Blot from one of the papers included in the landscape analysis was not cited among the serological techniques used, we have amended all relevant figures to include this data.**

**Conclusions**
Reviewer #1: The public health relevance is clearly addressed. they discussed how these data can be helpful for loiasis studies.

**REPLY: thank you, we are please that the message was conveyed appropriately**

Reviewer #2:

5. The influence of different patient groups (residents in endemic areas versus travellers) needs to be discussed, as it may affect the analytical performance of diagnostic tools for loasis.

**REPLY: we thank the reviewer for this observation. We actually considered the suggested analysis when we were designed the analysis plan. However, only six studies were conducted outside endemic areas with samples from individuals not obtained in endemic areas (three studies conducted outside endemic areas were actually analyzing samples obtained in endemic areas, as reported in the raw data file), and in these six studies, it was not possible to extract data separately for migrants (continuously exposed in endemic areas) and travelers (just exposed for a brief period). In addition, when looking at the comparisons of assays performed in the six studies performed outside endemic areas on samples obtained outside endemic areas, they were comparing different assays, and they were making up only one or two of the studies, assessing the same broad category comparison, made in endemic and non-endemic settings. For all these reasons we deemed not appropriate to carry out this sub-analysis formally. However, we agree with the reviewer that highlighting and discussing this aspect is important; therefore, we have made the following amendments to the paper: 1) we have amended the S5 and S6 files by indicating with an asterisk the papers who provide data from samples obtained outside endemic areas, so that the reader can clearly see where the results place in comparison with those of studies carried out on samples obtained in endemic areas and we have explicitly made a reference to this in the results (pp10 line 28-30 and following pp11 line 1-4; pp14 line 8-14); 2) we have added a sentence pointing out this distinction in the results when a trend difference could be identified (pp12 line 17-19 and pp16 line 28-29); 3) we have added a discussion point in the discussion (pp20 line 7-14).**

6. Is the significant and unexpected difference in sensitivity between the various microscopic diagnostic techniques really due solely to the type of blood sample? Could the populations studied as part of the evaluation of the analytical performance of the concentration techniques (occult loiasis/microfilariae) and the volumes of blood used have led to an underestimation of the sensitivity of these techniques ?

**REPLY: Indeed the surprising result was that sensitivity was similar when blood was examined in microliters vs concentrated milliliters. We actually (pp19 line 21-23) leave an open question as to whether this was explainable by the type of blood sample (capillary was venous) and actually point out that when a real head-to-head comparison of thick and mf concentration techniques was done, the latter was more sensitive, as also more intuitive and biologically plausible (pp19 line 25-30). We thank the reviewer for suggesting to expand the discussion of this result by adding the considerations on the populations/settings where the techniques were differently applied, which is now added in pp19 lines 23-25.**

**Editorial and Data Presentation Modifications?**
Reviewer #1: This study did not take into account an important work by Dieki et al" Profile of loiasis infection through clinical and laboratory diagnostics: the importance of biomarkers " publish in Transaction of Royal society of Trop Med in 2023 this study addressed specifically the problem for diagnostic of amicrofilaremic using western blot technique. this method is not mentioned in this manuscript.

**REPLY: we are deeply sorry for this overlook. We have amended both the S4 File, Table 1, Figure 2 and the landscape analysis paragraph (pp9 line 25) and following calculations, to include the western blot technique described in the paper, which was already included in the landscape analysis for the other assays used (microscopy, PCR and ELISA).**

Reviewer #2: Minor revisions:
1. Line 154: typing error “6) diagnostic test/s applied;”

**REPLY: amended**

2. Table 1: standardisation of the presentation of figures using dots or commas

**REPLY: thank you for having spotted these typos; amended.**

3. Line 347: typing error on “the reference “. The reference method is not a composite microscopy method in this case?

**REPLY: we have amended the typo and the reference test (for some reason the “plus PCR” was erased by mistake.**

4. Line 362 and 369: A redundant copy-and-paste phrase in relation to the rest of the text. In my view, this should be removed.

**REPLY: we have removed “when compared to direct diagnostic tests (microscopy, PCR) as a reference” to the second sentence indicated by the reviewer, to avoid the redundancy.**

5. Line 569 : typing error « for th ».

**REPLY: amended**

**Summary and General Comments**
Reviewer #1: this study review the available laboratory diagnostic test. Its evaluated their sensitivity and specificity by using the De-rSimonian Laird approach, and New castle- Ottawa scale methods. the search was perfomed on 1891 publications and end up with 99 selected. its came with the conclusion that ELISA and PCR are appropriate for screening while microscopic examination ( thick smear, leucoconcentration) remain important in any case of loiasis. Although the authors raised the question of amicrofilaremic or occult loiasis, they did not show how to diagnose this specific group which do not have circulating microfilaria in peripheral blood. ELISA detect antibodies which are not necessarly due to active infection. antibodies may reflect past contact with parasite. the other method suggested is PCR ,but this method is sign of existing microfilaria some where in the body not necessarly adult worm which is presents and can be seen during occular passage. In this context I suggest to authors to expand their study to the work performed by Dieki et al ( 2023)=Profile of loiasis infection through clinical and laboratory diagnostics: the importance of biomarkers Transactions of The Royal Society of Tropical Medicine and Hygiene, Volume 117, Issue 5.

**REPLY: we are pleased that the outcome message we wanted to convey from the review was clear, as nicely summarized here by the reviewer. As mentioned in the reply to the previous comment of the reviewer, the paper Dieki et al 2023 was indeed included in the landscape analysis, and we are again very sorry for having overlooked to mention the WB assay performed, which was now included in all related parts of the paper. Unfortunately, we could not include the data from this paper in the sensitivity and specificity formal analysis due to the difficulty in unequivocally extract or derive TP TN FP FN figures from the data presented in the text vs the tables and the imperfect adherence to case definition for inclusion that we envisaged for the review protocol of this part. However, we are thankful to the reviewer for having raised our attention again to this important paper and upon this hint we have amended the discussion (pp18 line 16-18 and line 29-30) by citing this study (now ref 58) and highlighting the important information obtained by the use of the WB.**

Reviewer #2: (No Response)
