# Supplementary material for: Laboratory diagnosis of loiasis to support individual patient management: A systematic review
Source: PLoS Negl Trop Dis. 2026 Jul 13;20(7):e0014460. doi: 10.1371/journal.pntd.0014460 (PMC13379093; doi:10.1371/journal.pntd.0014460)
Supplement: Peer review history

# Peer review: Decision Letter 0

**DOI:** 10.1371/journal.pntd.0014460.r001

**Authors:** Uriel Koziol, Bruce Rosa

15 May 2026

PNTD-D-26-00462

Laboratory diagnosis of loiasis to support individual patient management: a systematic review

PLOS Neglected Tropical Diseases

Dear Dr. Tamarozzi,

Thank you for submitting your manuscript to PLOS Neglected Tropical Diseases. After careful consideration, we feel that it has merit but does not fully meet PLOS Neglected Tropical Diseases's publication criteria as it currently stands. Therefore, we invite you to submit a revised version of the manuscript that addresses the points raised during the review process.

Please submit your revised manuscript within by Jul 14 2026 11:59PM. If you will need more time than this to complete your revisions, please reply to this message or contact the journal office at plosntds@plos.org. When you're ready to submit your revision, log on to https://www.editorialmanager.com/pntd/ and select the 'Submissions Needing Revision' folder to locate your manuscript file.

Please include the following items when submitting your revised manuscript:

* A letter that responds to each point raised by the editor and reviewer(s). You should upload this letter as a separate file labeled 'Response to Reviewers'. This file does not need to include responses to any formatting updates and technical items listed in the 'Journal Requirements' section below.

* A marked-up copy of your manuscript that highlights changes made to the original version. You should upload this as a separate file labeled 'Revised Manuscript with Track Changes'.

* An unmarked version of your revised paper without tracked changes. You should upload this as a separate file labeled 'Manuscript'.

If you would like to make changes to your financial disclosure, competing interests statement, or data availability statement, please make these updates within the submission form at the time of resubmission. Guidelines for resubmitting your figure files are available below the reviewer comments at the end of this letter.

As the corresponding author, your ORCID iD is verified in the submission system and will appear in the published article. PLOS supports the use of ORCID, and we encourage all coauthors to register for an ORCID iD and use it as well. Please encourage your coauthors to verify their ORCID iD within the submission system before final acceptance, as unverified ORCID iDs will not appear in the published article. Only the individual author can complete the verification step; PLOS staff cannot verify ORCID iDs on behalf of authors.

We look forward to receiving your revised manuscript.

Kind regards,

Bruce A. Rosa

Academic Editor

PLOS Neglected Tropical Diseases

Uriel Koziol

Section Editor

PLOS Neglected Tropical Diseases

Shaden Kamhawi

co-Editor-in-Chief

PLOS Neglected Tropical Diseases

orcid.org/0000-0003-4304-636XX

Paul Brindley

co-Editor-in-Chief

PLOS Neglected Tropical Diseases

orcid.org/0000-0003-1765-0002

**Additional Editor Comments:**

Please carefully consider the reviewer feedback, and add references and correct numbers as necessary.

**Journal Requirements:**

Please amend your detailed Financial Disclosure statement. This is published with the article. It must therefore be completed in full sentences and contain the exact wording you wish to be published.

1) State the initials, alongside each funding source, of each author to receive each grant. For example: "This work was supported by the National Institutes of Health (####### to AM; ###### to CJ) and the National Science Foundation (###### to AM)."

2) State what role the funders took in the study. If the funders had no role in your study, please state: "The funders had no role in study design, data collection and analysis, decision to publish, or preparation of the manuscript.".

If you did not receive any funding for this study, please simply state: u201cThe authors received no specific funding for this work.u201d

**Reviewers' Comments:**

Reviewer's Responses to Questions

**Key Review Criteria Required for Acceptance?**

As you describe the new analyses required for acceptance, please consider the following:

**Methods**

-Are the objectives of the study clearly articulated with a clear testable hypothesis stated?

-Is the study design appropriate to address the stated objectives?

-Is the population clearly described and appropriate for the hypothesis being tested?

-Is the sample size sufficient to ensure adequate power to address the hypothesis being tested?

-Were correct statistical analysis used to support conclusions?

-Are there concerns about ethical or regulatory requirements being met?

Reviewer #1: the objective is clearly articulated, the study design and statistical analysis used support the conclusion.

Reviewer #2: Loiasis is an endemic parasitic disease in Central and West Africa that is underestimated, despite significant associated mortality and morbidity in endemic areas. This disease has come to attract attention in ‘Northern’ countries due to the diagnosis of imported cases in non-endemic areas.

This condition remains poorly understood and difficult to diagnose, with available diagnostic tools offering variable performance. In this manuscript, the authors have conducted a review of the literature on the various diagnostic techniques for loiasis in order to better understand the different tools available and their analytical performance. The short-term objective is to develop guidelines for the individual management of loiasis

This work is of interest to the scientific community given the lack of knowledge about loiasis and the methods for diagnosing it. The authors’ objectives are clear, the methodology is clearly explained, and the tables and graphs are useful and easy to understand, and the manuscript is well written.

However, certain methodological aspects still need to be developed and some results need to be revised in order to clarify this work

Major revision:

1. Line 127: As stated by the authors, loiasis can present in various clinical and/or biological forms in infected patients. The aim of this study is to analyse all the diagnostic techniques used to detect Loa loa infection. However, the authors did not specify in the methodology what the definitions of Loa loa infection were in the various included studies (conjunctival passage of an adult, microfilaremia, positive serology +/- clinical symptoms +/- eosinophilia). Details of the various definitions used would provide a better understanding of the patient groups studied and put the results of the analytical performance of the different techniques into perspective

2. Line 176-186: The clinical and laboratory presentation of loiasis can vary depending on the patient population (https://doi.org/10.1093/infdis/163.6.1318), as recently reviewed (https://doi.org/10.1016/j.idnow.2025.105053). With a view to drafting future guidelines for the individual management of patients with loiasis, it is important to take into account the type of population, as the analytical performance of diagnostic tools may vary. Where possible, it would be useful to stratify analytical performance according to the study area: endemic (population continuously exposed and immunologically tolerant) and non-endemic (travellers with occasional exposure and low parasite load).

**********

**Results**

-Does the analysis presented match the analysis plan?

-Are the results clearly and completely presented?

-Are the figures (Tables, Images) of sufficient quality for clarity?

Reviewer #1: data are presented correctly

Reviewer #2: Major revision:

3. Line 220-301 and table 1: This paragraph is difficult to understand because there are numerous discrepancies between the figures given in the text and those in Table 1. There are also inconsistencies within Table 1 itself.

Line 227: microscopy was applied in 91/99 in the text but calculated as 96 in the table..?

Line 229: “73%” I don't understand where that figure comes from?

Line 237: leukoconcentration “32/35” in the text but estimated at 31 in the table.

Line 244: Loascope at “5/99” but written at 7/99 in the table?

Line 247 and 257: Discrepancies between text and table.

4. “Performances assessment: sensitivity” section: where possible, presenting the accuracy of diagnostic techniques according to the study area (endemic/non-endemic) would help to put the results presented into context.

**********

**Conclusions**

-Are the conclusions supported by the data presented?

-Are the limitations of analysis clearly described?

-Do the authors discuss how these data can be helpful to advance our understanding of the topic under study?

-Is public health relevance addressed?

Reviewer #1: The public health relevance is clearly addressed. they discussed how these data can be helpful for loiasis studies.

Reviewer #2: 5. The influence of different patient groups (residents in endemic areas versus travellers) needs to be discussed, as it may affect the analytical performance of diagnostic tools for loasis.

6. Is the significant and unexpected difference in sensitivity between the various microscopic diagnostic techniques really due solely to the type of blood sample? Could the populations studied as part of the evaluation of the analytical performance of the concentration techniques (occult loiasis/microfilariae) and the volumes of blood used have led to an underestimation of the sensitivity of these techniques ?

**********

**Editorial and Data Presentation Modifications?**

Use this section for editorial suggestions as well as relatively minor modifications of existing data that would enhance clarity. If the only modifications needed are minor and/or editorial, you may wish to recommend “Minor Revision” or “Accept”.

Reviewer #1: This study did not take into account an important work by Dieki et al" Profile of loiasis infection through clinical and laboratory diagnostics: the importance of biomarkers " publish in Transaction of Royal society of Trop Med in 2023 this study addressed specifically the problem for diagnostic of amicrofilaremic using western blot technique. this method is not mentioned in this manuscript.

Reviewer #2: Minor revisions:

1. Line 154: typing error “6) diagnostic test/s applied;”

2. Table 1: standardisation of the presentation of figures using dots or commas

3. Line 347: typing error on “the reference “. The reference method is not a composite microscopy method in this case?

4. Line 362 and 369: A redundant copy-and-paste phrase in relation to the rest of the text. In my view, this should be removed

5. Line 569 : typing error « for th »

**********

**Summary and General Comments**

Use this section to provide overall comments, discuss strengths/weaknesses of the study, novelty, significance, general execution and scholarship. You may also include additional comments for the author, including concerns about dual publication, research ethics, or publication ethics. If requesting major revision, please articulate the new experiments that are needed.

Reviewer #1: this study review the available laboratory diagnostic test. Its evaluated their sensitivity and specificity by using the De-rSimonian Laird approach, and New castle- Ottawa scale methods. the search was perfomed on 1891 publications and end up with 99 selected. its came with the conclusion that ELISA and PCR are appropriate for screening while microscopic examination ( thick smear, leucoconcentration) remain important in any case of loiasis. Although the authors raised the question of amicrofilaremic or occult loiasis, they did not show how to diagnose this specific group which do not have circulating microfilaria in peripheral blood. ELISA detect antibodies which are not necessarly due to active infection. antibodies may reflect past contact with parasite. the other method suggested is PCR ,but this method is sign of existing microfilaria some where in the body not necessarly adult worm which is presents and can be seen during occular passage. In this context I suggest to authors to expand their study to the work performed by Dieki et al ( 2023)=Profile of loiasis infection through clinical and laboratory diagnostics: the importance of biomarkers Transactions of The Royal Society of Tropical Medicine and Hygiene, Volume 117, Issue 5.

Reviewer #2: (No Response)

**********

PLOS authors have the option to publish the peer review history of their article (what does this mean?). If published, this will include your full peer review and any attached files.

If you choose “no”, your identity will remain anonymous but your review may still be made public.

**Do you want your identity to be public for this peer review?**  For information about this choice, including consent withdrawal, please see our Privacy Policy.

Reviewer #1: No

Reviewer #2: No

[NOTE: If reviewer comments were submitted as an attachment file, they will be attached to this email and accessible via the submission site. Please log into your account, locate the manuscript record, and check for the action link "View Attachments". If this link does not appear, there are no attachment files.]

**Figure resubmission:**

While revising your submission, we strongly recommend that you use PLOS’s NAAS tool (https://ngplosjournals.pagemajik.ai/artanalysis) to test your figure files. NAAS can convert your figure files to the TIFF file type and meet basic requirements (such as print size, resolution), or provide you with a report on issues that do not meet our requirements and that NAAS cannot fix.

After uploading your figures to PLOS’s NAAS tool - https://ngplosjournals.pagemajik.ai/artanalysis, NAAS will process the files provided and display the results in the "Uploaded Files" section of the page as the processing is complete. If the uploaded figures meet our requirements (or NAAS is able to fix the files to meet our requirements), the figure will be marked as "fixed" above. If NAAS is unable to fix the files, a red "failed" label will appear above. When NAAS has confirmed that the figure files meet our requirements, please download the file via the download option, and include these NAAS processed figure files when submitting your revised manuscript.

**Reproducibility:**

To enhance the reproducibility of your results, we recommend that authors of applicable studies deposit laboratory protocols in protocols.io, where a protocol can be assigned its own identifier (DOI) such that it can be cited independently in the future. Additionally, PLOS ONE offers an option to publish peer-reviewed clinical study protocols. Read more information on sharing protocols at https://plos.org/protocols?utm_medium=editorial-email&utm_source=authorletters&utm_campaign=protocols

# Author comment: Author response to Decision Letter 1

**DOI:** 10.1371/journal.pntd.0014460.r002

22 May 2026

Attachment Submitted filename: Feedback and replies.docx

# Peer review: Decision Letter 1

**DOI:** 10.1371/journal.pntd.0014460.r003

**Authors:** Uriel Koziol, Bruce Rosa, Uriel Koziol, Bruce Rosa

11 Jun 2026

Dear Dr Tamarozzi,

We are pleased to inform you that your manuscript 'Laboratory diagnosis of loiasis to support individual patient management: a systematic review' has been provisionally accepted for publication in PLOS Neglected Tropical Diseases.

Before your manuscript can be formally accepted you will need to complete some formatting changes, which you will receive in a follow up email. A member of our team will be in touch with a set of requests.

Please note that your manuscript will not be scheduled for publication until you have made the required changes, so a swift response is appreciated.

IMPORTANT: The editorial review process is now complete. PLOS will only permit corrections to spelling, formatting or significant scientific errors from this point onwards. Requests for major changes, or any which affect the scientific understanding of your work, will cause delays to the publication date of your manuscript.

Should you, your institution's press office or the journal office choose to press release your paper, you will automatically be opted out of early publication. We ask that you notify us now if you or your institution is planning to press release the article. All press must be co-ordinated with PLOS.

Thank you again for supporting Open Access publishing; we are looking forward to publishing your work in PLOS Neglected Tropical Diseases.

Best regards,

Bruce A. Rosa

Academic Editor

PLOS Neglected Tropical Diseases

Uriel Koziol

Section Editor

PLOS Neglected Tropical Diseases

Shaden Kamhawi

co-Editor-in-Chief

PLOS Neglected Tropical Diseases

orcid.org/0000-0003-4304-636XX

Paul Brindley

co-Editor-in-Chief

PLOS Neglected Tropical Diseases

orcid.org/0000-0003-1765-0002

***********************************************************

The authors have sufficiently addressed all reviewer concerns

p.p1 {margin: 0.0px 0.0px 0.0px 0.0px; line-height: 16.0px; font: 14.0px Arial; color: #323333; -webkit-text-stroke: #323333}span.s1 {font-kerning: none

Reviewer's Responses to Questions

**Key Review Criteria Required for Acceptance?**

As you describe the new analyses required for acceptance, please consider the following:

**Methods**

-Are the objectives of the study clearly articulated with a clear testable hypothesis stated?

-Is the study design appropriate to address the stated objectives?

-Is the population clearly described and appropriate for the hypothesis being tested?

-Is the sample size sufficient to ensure adequate power to address the hypothesis being tested?

-Were correct statistical analysis used to support conclusions?

-Are there concerns about ethical or regulatory requirements being met?

Reviewer #1: the study design is appropriate to address the stated objectives

**********

**Results**

-Does the analysis presented match the analysis plan?

-Are the results clearly and completely presented?

-Are the figures (Tables, Images) of sufficient quality for clarity?

Reviewer #1: the results, tables and figures are fine

**********

**Conclusions**

-Are the conclusions supported by the data presented?

-Are the limitations of analysis clearly described?

-Do the authors discuss how these data can be helpful to advance our understanding of the topic under study?

-Is public health relevance addressed?

Reviewer #1: the previous review comment on the original paper has been added to the paper. so no comment again

**********

**Editorial and Data Presentation Modifications?**

Use this section for editorial suggestions as well as relatively minor modifications of existing data that would enhance clarity. If the only modifications needed are minor and/or editorial, you may wish to recommend “Minor Revision” or “Accept”.

Reviewer #1: the authors have taken into account previous reviewer's comments . therefore I will recommend to Accept the paper in this form

**********

**Summary and General Comments**

Use this section to provide overall comments, discuss strengths/weaknesses of the study, novelty, significance, general execution and scholarship. You may also include additional comments for the author, including concerns about dual publication, research ethics, or publication ethics. If requesting major revision, please articulate the new experiments that are needed.

Reviewer #1: no comment

**********

PLOS authors have the option to publish the peer review history of their article (what does this mean?). If published, this will include your full peer review and any attached files.

If you choose “no”, your identity will remain anonymous but your review may still be made public.

**Do you want your identity to be public for this peer review?**  For information about this choice, including consent withdrawal, please see our Privacy Policy.

Reviewer #1: No

# Editor report: Acceptance letter

**DOI:** 10.1371/journal.pntd.0014460.r004

**Authors:** Uriel Koziol, Bruce Rosa, Uriel Koziol, Bruce Rosa

Dear Dr Tamarozzi,

We are delighted to inform you that your manuscript, "Laboratory diagnosis of loiasis to support individual patient management: a systematic review," has been formally accepted for publication in PLOS Neglected Tropical Diseases.

We have now passed your article onto the PLOS Production Department who will complete the rest of the publication process. All authors will receive a confirmation email upon publication.

The corresponding author will soon be receiving a typeset proof for review, to ensure errors have not been introduced during production. Please review the PDF proof of your manuscript carefully, as this is the last chance to correct any scientific or type-setting errors. Please note that major changes, or those which affect the scientific understanding of the work, will likely cause delays to the publication date of your manuscript. Note: Proofs for Front Matter articles (Editorial, Viewpoint, Symposium, Review, etc...) are generated on a different schedule and may not be made available as quickly.

Soon after your final files are uploaded, the early version of your manuscript will be published online unless you opted out of this process. The date of the early version will be your article's publication date. The final article will be published to the same URL, and all versions of the paper will be accessible to readers.

For Research Articles, you will receive an invoice from PLOS for your publication fee after your manuscript has reached the completed accept phase. If you receive an email requesting payment before acceptance or for any other service, this may be a phishing scheme. Learn how to identify phishing emails and protect your accounts at https://explore.plos.org/phishing.

Thank you again for supporting open-access publishing; we are looking forward to publishing your work in PLOS Neglected Tropical Diseases.

Best regards,

Shaden Kamhawi

co-Editor-in-Chief

PLOS Neglected Tropical Diseases

Paul Brindley

co-Editor-in-Chief

PLOS Neglected Tropical Diseases
